# Supplementary material for: Biomechanical risk factors for knee osteoarthritis and lower back pain in lower limb amputees: protocol for a systematic review
Source: BMJ Open. 2022 Nov 21;12(11):e066959. doi: 10.1136/bmjopen-2022-066959 (PMC9680152; doi:10.1136/bmjopen-2022-066959)
Supplement: Supplementary data [file bmjopen-2022-066959supp001.pdf]

Appendix 1: Outcome variable to be extracted from experimental studies

**Temporospatial**	**Kinematics**		**Kinetics**	
BMI	Knee adduction range of motion (ROM)		Peak knee adduction moment (KAM)	
Walking speed	Knee sagittal ROM		KAM loading rate (rate of force development)	
Stride width (from midline)	Hip-knee-ankle adduction angle		KAM impulse	
Stride/step length/symmetry	Varus/valgus angle - knee adduction angle		Peak knee joint contact forces	
Stance/contact time	Knee flexion at heel strike		Joint reaction force at terminal stance	
Leg length discrepancy	Knee flexion at toe off		Peak knee sagittal/flexion/extension plane moments	
Cadence		Knee	Knee rotation moment early stance	
			Knee flexion moment at loading response	
**Ground Reaction Force (GRF)**	Peak hip extension		Flexion moment at terminal stance	
Vertical GRF at heel strike	Peak hip flexion angle		Net work	
Horizontal GRF at heel strike	Hip flexion at toe off		Positive work	
Vertical GRF loading rate	Hip ROM sagittal		Negative work	Knee
Prosthetic horizontal GRF at push-off	Hip flexion at loading response	Hip		
Peak vertical GRF			Peak hip extension moment	Hip
	Peak trunk flexion angle			
	Peak lumbar spine extension		Lumbar-pelvic lateral joint reaction force	
	Lumbar-pelvis spine extension		Anterior lateral joint reaction force - lumbar-pelvic	
	Peak coronal/frontal/lateral/contralateral pelvic tilt		Mediolateral shear joint reaction force of trunk	
	Peak anterior-posterior/sagittal pelvic tilt		Anterior-posterior shear joint reaction force of trunk	
	Pelvic ROM in frontal plane		Compression joint reaction force forces of trunk	
	Pelvic ROM in sagittal plane		Lumbar/pelvic joint power	
	Lumbar transverse plane rotation ROM		Lower back joint contact force	
	Sagittal plane pelvis angle		Joint work L5/S1 (frontal and sagittal plane)	
	Side flexion of trunk-pelvis			Trunk/Pelvis
	Mediolateral trunk sway			
	Lumbar lordosis angle	Trunk/Pelvis		
