# Supplementary material for: Biomechanical risk factors for knee osteoarthritis and lower back pain in lower limb amputees: protocol for a systematic review
Source: BMJ Open. 2022 Nov 21;12(11):e066959. doi: 10.1136/bmjopen-2022-066959 (PMC9680152; doi:10.1136/bmjopen-2022-066959)
Supplement: Supplementary data [file bmjopen-2022-066959supp002.pdf]

Appendix 2: Search strategies for each of the five databases used in this systematic review	
Database	keywords
Web of Science	TS=("transtibial amput*" OR "transfemoral amput*" OR amput* OR "Lower limb amput*" OR "Lower extremity amput*" OR "Leg prosthesis") AND TS=(walking OR running OR gait OR locomotion OR biomechanics OR kinematics OR kinetics OR "biomechanical parameter*" OR *symmetr* OR forc* OR angle* OR moment* OR power EMG OR electromyogra*) AND TS=(Osteoporosis OR Osteopenia OR "Back Pain" OR Backache OR Osteoarthritis OR "musculoskeletal diseas*" OR "musculoskeletal condition*" OR "secondary diseas*")
Scopus	(TITLE-ABS-KEY ("transtibial amput*" OR "transfemoral amput*" OR amput* OR "Lower limb amput*" OR "Lower extremity amput*" OR "Leg prosthesis") AND TITLE-ABS-KEY (walking OR running OR gait OR locomotion OR biomechanics OR kinematics OR kinetics OR "biomechanical parameter*" OR *symmetr* OR forc* OR angle* OR moment* OR power OR emg OR electromyogra*) AND TITLE-ABS-KEY (osteoporosis OR osteopenia OR "Back Pain" OR backache OR osteoarthritis OR "musculoskeletal diseas*" OR "musculoskeletal condition*" OR "secondary diseas*")) AND (LIMIT-TO (LANGUAGE , "English") OR LIMIT-TO (LANGUAGE , "German") OR LIMIT-TO (LANGUAGE , "Italian"))
Pubmed	((("Amputees"[Mesh] OR "Amputation"[Mesh] OR Amput* OR "Amputation, Traumatic"[Mesh] OR "Amputation, Congenital" [Supplementary Concept] OR "lower limb amputation" OR "lower limb amputee" OR "lower extremity amputee" OR “Artificial Limb”[Mesh]) AND ("Walking"[Mesh] OR “Running”[Mesh] OR “Gait”[Mesh] OR gait OR “Locomotion”[Mesh] OR Locomotion OR “Biomechanical Phenomena”[Mesh] OR biomechanic* OR "biomechanical parameter*" OR symmetr* OR angle OR angles OR force OR “Ground Reaction forces” OR power OR “kinetics”[Mesh] OR “Kinematics”[Mesh] OR kinetic* Or kinematic* OR EMG) AND (“Osteoarthritis”[Mesh] OR “osteoporosis”[Mesh] OR osteopenia OR “Back Pain”[Mesh] OR backache OR “musculoskeletal disease*”[Mesh] OR “secondary diseas*” OR “secondary condition” OR “knee osteoarthritis” OR “hip osteoarthritis”)))
Embase	Option A<br>('transtibial amputation'/exp OR 'transtibial amputation' OR 'transfemoral amputation'/exp OR 'transfemoral amputation' OR 'amputee'/exp OR 'amputee' OR 'amputees'/exp OR 'amputees' OR 'individual with amputation'/exp OR 'individual with amputation' OR 'person with amputation'/exp OR 'person with amputation' OR 'artificial leg'/exp OR 'artificial leg' OR 'artificial legs'/exp OR 'artificial legs' OR 'leg prostheses'/exp OR 'leg prostheses' OR 'leg prosthesis'/exp OR 'leg prosthesis' OR 'leg prosthetics'/exp OR 'leg prosthetics' OR 'leg, artificial'/exp OR 'leg, artificial' OR 'legs, artificial'/exp OR 'legs, artificial' OR 'lower extremity prosthesis'/exp OR 'lower extremity prosthesis' OR 'lower limb prostheses'/exp OR 'lower limb prostheses' OR 'lower limb prosthesis'/exp OR 'lower limb prosthesis' OR 'prostheses, leg'/exp OR 'prostheses, leg' OR 'prosthesis, leg'/exp OR 'prosthesis, leg' OR 'walking prosthesis'/exp OR 'walking prosthesis' OR 'amputation, traumatic'/exp OR 'amputation, traumatic' OR 'traumatic amputation'/exp OR

	'traumatic amputation' OR 'congenital amputation'/exp OR 'congenital amputation') AND ('walking'/exp OR 'walking' OR 'runner'/exp OR 'runner' OR 'running'/exp OR 'running' OR 'kinematics'/exp OR 'kinematics' OR 'human kinetics'/exp OR 'human kinetics' OR 'kinetic analysis'/exp OR 'kinetic analysis' OR 'kinetic mechanism'/exp OR 'kinetic mechanism' OR 'kinetic model'/exp OR 'kinetic model' OR 'kinetics'/exp OR 'kinetics' OR 'biomechanical phenomena'/exp OR 'biomechanical phenomena' OR 'biomechanical phenomenon'/exp OR 'biomechanical phenomenon' OR 'biomechanics'/exp OR 'biomechanics' OR 'biomechanism'/exp OR 'biomechanism' OR 'behavior, locomotor'/exp OR 'behavior, locomotor' OR 'behaviour, locomotor'/exp OR 'behaviour, locomotor' OR 'locomotion'/exp OR 'locomotion' OR 'locomotion pattern'/exp OR 'locomotion pattern' OR 'locomotor activity'/exp OR 'locomotor activity' OR 'locomotor response'/exp OR 'locomotor response' OR 'biped gait'/exp OR 'biped gait' OR 'gait'/exp OR 'gait' OR 'gait analysis'/exp OR 'gait analysis' OR 'gait training'/exp OR 'gait training' OR 'pattern, walking'/exp OR 'pattern, walking' OR 'walking pattern'/exp OR 'walking pattern') AND ('decalcification, pathologic'/exp OR 'decalcification, pathologic' OR 'endocrine osteoporosis'/exp OR 'endocrine osteoporosis' OR 'osteoporosis'/exp OR 'osteoporosis' OR 'osteoporotic decalcification'/exp OR 'osteoporotic decalcification' OR 'osteoarthritis'/exp OR 'osteoarthritis' OR 'back ache'/exp OR 'back ache' OR 'back pain'/exp OR 'back pain' OR 'back pain syndrome'/exp OR 'back pain syndrome' OR 'backache'/exp OR 'backache' OR 'backpain'/exp OR 'backpain' OR 'dorsalgia'/exp OR 'dorsalgia' OR 'pain, back'/exp OR 'pain, back' OR 'musculoskeletal disease'/exp OR 'musculoskeletal disease' OR 'secondary disease'/exp OR 'secondary disease')
CINAHL	OPTION A: Straight search of terms (no subheading selection)<br><br>("transtibial amput*" OR "transfemoral amput*" OR amput* OR "Lower limb amput*" OR "Lower extremity amput*" OR "Leg prosthesis")<br><br>AND<br><br>("Walking" OR “Running” OR “Gait” OR gait OR “Locomotion” OR Locomotion OR “Biomechanical Phenomena” OR biomechanic* OR "biomechanical parameter*" OR symmetr* OR angle OR angles OR force OR “Ground Reaction forces” OR power OR “kinetics” OR “Kinematics” OR kinetic* Or kinematic OR "EMG" OR electromyo*)<br><br>AND<br><br>(“Osteoarthritis” OR “osteoporosis” OR osteopenia OR “Back Pain” OR backache OR “musculoskeletal disease*” OR “secondary diseas*” OR “secondary condition” OR “knee osteoarthritis” OR “hip osteoarthritis”)
