# Supplementary material for: Biomechanical risk factors for knee osteoarthritis and lower back pain in lower limb amputees: protocol for a systematic review
Source: BMJ Open. 2022 Nov 21;12(11):e066959. doi: 10.1136/bmjopen-2022-066959 (PMC9680152; doi:10.1136/bmjopen-2022-066959)
Supplement: Author's manuscript [file bmjopen-2022-066959.draft_revisions.pdf]

BMJ Open

BMJ Open is committed to open peer review. As part of this commitment we make the peer review history of every article we publish publicly available.

When an article is published we post the peer reviewers' comments and the authors' responses online. We also post the versions of the paper that were used during peer review. These are the versions that the peer review comments apply to.

The versions of the paper that follow are the versions that were submitted during the peer review process. They are not the versions of record or the final published versions. They should not be cited or distributed as the published version of this manuscript.

BMJ Open is an open access journal and the full, final, typeset and author-corrected version of record of the manuscript is available on our site with no access controls, subscription charges or pay-per-view fees (http://bmjopen.bmj.com).

If you have any questions on BMJ Open's open peer review process please email info.bmjopen@bmj.com

BMJ Open

Biomechanical risk factors for knee osteoarthritis and lower back pain in lower limb amputees: protocol for a systematic review

Journal:	*BMJ Open*
Manuscript ID	bmjopen-2022-066959
Article Type:	Protocol
Date Submitted by the Author:	28-Jul-2022
Complete List of Authors:	Wade, Logan; University of Bath, Department for Health<br>McGuigan, M. Polly; University of Bath, Department for Health<br>McKay, Carly; University of Bath, Department for Health<br>Bilzon, James; University of Bath, Department for Health<br>Seminati, Elena; University of Bath, Department for Health
Keywords:	Diabetic nephropathy & vascular disease < DIABETES & ENDOCRINOLOGY, REHABILITATION MEDICINE, SPORTS MEDICINE

SCHOLARONE™
Manuscripts

BIOMECHANICAL RISK FACTORS FOR KNEE OSTEOARTHRITIS AND LOWER BACK PAIN IN LOWER LIMB AMPUTEES: PROTOCOL FOR A SYSTEMATIC REVIEW

Logan Wade[a,b], M. Polly McGuigan[a,b], Carly McKay[a,c,d], James Bilzon[a,b,d], Elena Seminati[a,b]

a) Department for Health, University of Bath, Bath UK

b) Centre for Analysis of Motion, Entertainment Research and Applications, University of Bath, United Kingdom

c) Centre for Health and Illness and Injury Prevention in Sport, University of Bath, Bath UK

d) Centre for Sport Exercise and Osteoarthritis Research Versus Arthritis, University of Bath, Bath UK

Corresponding Author:

Logan Wade

lw2175@bath.ac.uk

University of Bath

1 West, Office 5.111, Bath, BA2 7AY, United Kingdom

Email:

Logan Wade - lw2175@bath.ac.uk, M. Polly McGuigan - mpm21@bath.ac.uk, Carly McKay - cdm47@bath.ac.uk, James Bilzon - jb438@bath.ac.uk, Elena Seminati - es685@bath.ac.uk

Registration

In accordance with guidelines, this protocol was submitted and approved with the International Prospective Register of Systematic reviews (PROSPERO) on 03/02/2020 and was last updated 21/01/2022 (ID: CRD42020158247).

For peer review only - http://bmjopen.bmj.com/site/about/guidelines.xhtml

Abstract

Introduction: There is limited research exploring biomechanical risk factors for the development of knee osteoarthritis (KOA) and lower back pain (LBP) between lower limb amputee sub-groups, [e.g., transtibial amputees (TTA) vs transfemoral amputees (TFA) or TTA dysvascular vs TTA traumatic]. Previous reviews have focussed primarily on studies where symptoms of KOA or LBP are present, however, due to limited study numbers, this hinders their scope and ability to compare between amputee sub-groups. Therefore, the aim of this systematic review is to descriptively compare biomechanical risk factors for developing KOA and LBP between lower limb amputee sub-groups, irrespective of whether KOA or LBP was present.

Methods and analysis: This review is currently in progress and screening results are presented alongside the protocol to highlight challenges encountered during data extraction. Five electronic databases were searched (Medline – Web of Science, PubMed, CINAHL, Embase and Scopus). Eligible studies were observational or interventional, reporting biomechanical gait outcomes for individual legs in adult lower limb amputees during flat walking, incline/decline walking or stair ascent/descent. Two reviewers screened for eligibility and level of agreement was assessed using Cohen's Kappa. Data extraction is ongoing. Risk of bias will be assessed using a modified Downs and Black method, and outcome measures will be descriptively synthesised.

Ethics and dissemination: There are no ethical considerations for this systematic review. Due to its scope, results are expected to be published in three separate manuscripts: 1) Biomechanical risk factors of KOA between TTA and TFA, relative to non-amputees, 2) Biomechanical risk factors of LBP between TTA and TFA, relative to non-amputees, and 3) Biomechanical risk factors of KOA and LBP between transtibial amputees with traumatic or dysvascular causes, relative to non-amputees.

PROSPERO registration number: CRD42020158247).

For peer review only - http://bmjopen.bmj.com/site/about/guidelines.xhtml

Strength and Limitations

- This systematic review protocol follows the Preferred Reporting Items for Systematic Reviews and Meta-Analyses Protocols (PRISMA-P) guidelines.
- This will be the first review to compare biomechanical gait between amputee sub-groups such as transtibial and transfemoral amputees, as well as transtibial dysvascular and transtibial traumatic amputees.
- Studies must include at least one temporospatial, joint kinematic or joint kinetic outcome measure for individual legs to ensure valid and thorough biomechanical technique and analysis.
- Unexpected variations in measurement methodologies have required papers without non-amputee controls to be removed from the study, to ensure rigorous comparison between amputee sub-groups.

Introduction

Lower limb amputations of the hip, knee and ankle considerably alter walking gait and function, with over 42 000 major lower limb amputations performed over a ten year period (2003-2013) in the UK [1]. In 2005, major lower limb amputations in the USA and UK accounted for over 90% of all major limb amputations [2, 3] and compared to healthy populations, lower-limb amputees have significantly higher rates of secondary disorders such as knee osteoarthritis [4, 5] and lower back pain [6-11]. These higher rates are likely due to altered gait mechanics that are experienced as a result of missing limbs and joints [12], requiring the intact leg to support greater load and produce increased force to maintain stable gait. Furthermore, differences between amputation levels (below ankle, below knee and above knee) and amputation causes (traumatic, vascular disease, cancer, congenital) likely produce different functional impairments, which may increase risk of developing knee osteoarthritis (KOA) and lower back pain (LBP).

For peer review only - http://bmjopen.bmj.com/site/about/guidelines.xhtml

Considering the prevalence of lower limb amputations, transfemoral (above the level of the knee) amputees (TFA) and through knee (at the level of the knee joint) amputees account for 17-23% of all amputations [13, 14]. Transtibial (below the level of the knee) amputees (TTA) and through ankle (at the level of the ankle joint) amputees account for 12-32%, while partial foot amputees account for 15-26% of all amputations [13, 14]. Minor amputations of the foot make up the remaining percentages, however these generally do not substantially alter gait and are therefore not a focus of this review. As amputation level moves up the leg, functional mobility and quality of life is reduced [15], requiring greater altered gait mechanics to accommodate the limited power output and instability of the prosthetic limb during stance [12]. Thus, above knee amputees are at an increased risk of developing knee pain [4] and KOA in the intact limb compared to below knee amputees, with OA of the intact knee occurring in roughly 60% of TFAs and 40% of TTAs, compared to just 20% of non-amputees [16]. Similarly, prevalence of LBP is found in roughly 50-76% of lower limb amputees, compared to 35% of non-amputees [10-12]. Evidence suggests that there may not be a difference in prevalence or intensity of LBP between TTA and TFA [17], although a previous systematic review of LBP in lower limb amputees was unable to draw comparisons between TTA and TFA due to limited studies in TTA [18]. Thus, there is a need to explore biomechanical gait differences between TTAs and TFAs, to understand how biomechanical risk factors associated with the development of and potential predisposition to KOA and LBP differ between groups.

While amputation level plays a crucial role in altered gait mechanics, cause of amputation likely also contributes significantly to the development of secondary musculoskeletal symptoms. The two primary causes of amputation are vascular diseases and traumatic accidents, with cancer and congenital causes only making up 1-3% of all amputations [3, 14]. Prevalence of amputation cause varies worldwide, with traumatic amputations making up 6 - 45% of all amputations [3, 14] and patients primarily characterised as being young and fit [3]. Alternatively, dysvascular amputations have increased significantly in recent decades due to the increasing prevalence of diabetes and dysvascular disease, making up 65%-91% of all amputations [3, 14]. This population is generally older than other amputee cause types [3] and commonly have a higher body mass index [19], which additionally puts individuals at a greater risk of KOA [20]. Dysvascular

For peer review only - http://bmjopen.bmj.com/site/about/guidelines.xhtml

amputees also have poorer uptake of prosthetic devices, which further increases their risk of sedentary lifestyle and weight gain after amputation [21]. Counterintuitively, some research suggests that this lower activity status and prosthetic use may result in TFAs having a reduced risk of developing LBP compared to traumatic amputees [16, 18]. Unfortunately, despite a much higher prevalence of dysvascular amputations, gait biomechanics research within this population is relatively limited, especially compared to the high proportion of research surrounding traumatic amputations [4, 11, 18, 22-25]. We therefore need to determine whether current research, investigating the development of secondary disorders primarily in traumatic amputees, is generalisable to dysvascular amputees, and if there are any additional biomechanical factors specific to dysvascular amputees that would increase or decrease their likelihood of developing KOA and LBP.

Additional sub-groups include bi-lateral amputees, osseo-integrated amputees and adult amputees who had an amputation as children or were born without a limb (i.e. congenital amputees). Bilateral amputees have a high variation between individuals, often presenting with multiple amputation levels (e.g. one leg with a trans-tibial amputation and the other with a trans-femoral amputation), which can dramatically alter gait and may influence development of secondary disorders. Osseo-integrated amputees generally do not suffer from skin problems, ill-fitting prosthesis issues or bone degeneration issues of their socket wearing counterparts. Thus, this population may have greater prosthetic use and increased risk of KOA and LBP, although they also have alternate complications such a recurring infections and risk of bone fractures [26, 27]. Finally, adult amputees who experienced amputations during childhood, or were congenital amputees, have spent the most time with their amputation. This group may have altered gait patterns as a function of growing with their prosthesis, which may place them at an increased risk of developing secondary symptoms much earlier in life. Across all amputee sub-groups, the primary barrier to understanding altered biomechanical gait is in recruiting a sufficient sample from each population, especially in these latter specialised sub-groups. Furthermore, longitudinal cohort studies, following patients throughout their life are very rare, with most studies being performed cross-sectionally. Therefore, a large-scale systematic review that examines biomechanical gait between amputee sub-groups is presently the best available

For peer review only - http://bmjopen.bmj.com/site/about/guidelines.xhtml

option for exploring which biomechanical gait factors may contribute to development of KOA or LBP between lower limb amputee populations.

Several reviews have examined amputee biomechanical gait with a focus on KOA and LBP. However, the majority of these reviews have not been performed using systematic methods [11, 22, 23, 28-30], and generally have not described differences between amputee sub-groups, often only including a single sub-group (e.g. only traumatic or transtibial amputees). Moreover, those few systematic reviews on gait and secondary disorders in amputees have generally only been performed on a single amputee subgroup, using studies where symptoms of KOA or LBP are present, which severely limits their scope (11-17 studies per review) and ability to compare between amputee groups [16, 18, 31, 32]. Due to such small study numbers included within these systematic reviews, knowledge of the biomechanical gait characteristics associated with KOA and LBP and their prevalence between amputee sub-groups is considerably limited. Sagawa, Turcot [33] has performed a large-scale systematic review (89 studies) of altered biomechanical gait factors across all lower limb amputees, aiming to broadly characterise biomechanics and physiological parameters during gait. They identified that TTA knee flexion during heel strike is limited to 9-12˚, while TFA knee flexion was zero or negative (extension). Additionally, TFAs had twice the pelvic ROM compared to healthy individuals which may contribute to the development of LBP. Unfortunately, their review was very broad, was not targeted at gait characteristics of KOA and LBP and generally did not make any comparisons or conclusions between sub-groups (e.g., amputation level or amputation cause). To fill this gap in the literature, a large-scale systematic review targeted at identifying how biomechanical risk factors of KOA and LBP differ between amputee sub-groups is needed. Understanding what biomechanical factors influence gait will help facilitate specific and personalised rehabilitation programmes and prosthetic designs.

OBJECTIVES

While previous systematic reviews have been limited by only including studies with amputees who are diagnosed with KOA and LBP, there is a substantial amount of experimental literature that has examined

For peer review only - http://bmjopen.bmj.com/site/about/guidelines.xhtml

lower limb amputee gait and posture where no KOA or LBP has been recorded. Because of the high prevalence of KOA and LBP, it is likely that biomechanical abnormalities leading to these secondary disorders will be present across the majority of amputees. Therefore, the aim of this systematic review is to descriptively compare biomechanical risk factors for developing KOA and LBP between amputee sub-groups, irrespective of whether KOA or LBP was present. Amputee sub-groups will be categorised by level of amputation (below ankle, below knee and above knee), cause of amputation (vascular disease, traumatic injury, cancer, congenital) and special sub-groups (bilateral amputees, osseo-integrated amputees, adult amputees who had an amputation or congenital missing limb as children. Individual sub-groups will only be included for analysis if sufficient data is available to support comparisons (see data extraction section).

Methods

This systematic review is currently in progress and screening results are presented within this paper to highlight challenges encountered during data extraction. This approach was chosen to ensure the transparency of our methods and increase the replicability of the review.

ELIGIBILITY CRITERIA

In accordance with PRISMA-P guidelines [34], this protocol was submitted and approved by the International Prospective Register of Systematic reviews (PROSPERO) on 03/02/2020 and was last updated 21/01/2022 (ID: CRD42020158247). This protocol has adhered to the PRIMSA-P guide and checklist for publishing systematic review protocols [34].

STUDY CHARACTERISTICS

Studies included in this review had to be observational studies such as cross-sectional/cohort studies and longitudinal studies. Intervention and randomised control trial (RCT) studies were included in this review but only the control amputee group or baseline measures were extracted (observational data). Review

For peer review only - http://bmjopen.bmj.com/site/about/guidelines.xhtml

papers, case studies, conference proceedings and animal studies were excluded. Studies that included quantitative biomechanical measures of lower limb amputees were included if results were reported for individual legs (intact leg and prosthetic leg presented separately). To ensure application of valid and thorough biomechanical technique and analysis, data had to include at least one temporospatial, joint kinematic or joint kinetic outcome measure for individual legs (see Appendix 2 for a full list of extracted outcome measures). Outcome variables were determined from previous reviews that outlined biomechanical differences between: amputees and non-amputee populations [12, 17, 22, 23, 28, 33, 35]; healthy non-amputee populations and KOA and LBP non-amputee populations [36-38]; and healthy amputees and amputees with KOA and LBP [12, 16, 18, 31, 32]. While ground reaction force (GRF) outcome measures for individual strides were extracted, studies that only reported GRF measures were not included in this review, as GRF is a measure of full body force and is not specific to the knee joint or lower back region. Observational studies had to be performed during walking on flat, incline or stair surfaces, at either preferred or controlled walking speeds. Studies that only investigated running-specific prostheses or running gaits were not included. Studies that examined powered ankles were included in this review, but only if an unpowered condition was performed. All microprocessor-controlled ankles and knees (devices that do not add energy to the system) were included in this review.

PARTICIPANTS

Lower limb amputees were included in this review, but only if results were separated by different amputation levels (e.g., studies that combined results of transtibial and transfemoral amputees were not included). Due to the differences between child and adult gait, and the focus on development of secondary disorders which primarily occurs in adults, studies performed only on children (younger than 18 years) were not included.

Patient and Public Involvement

None

For peer review only

For peer review only - http://bmjopen.bmj.com/site/about/guidelines.xhtml

INFORMATION SOURCES

Literature searches were performed across five databases: Medline – Web of Science, PubMed, CINAHL, Embase and Scopus. Manual searches were conducted using the reference lists within previous reviews and reference lists within papers obtained from database searches, to ensure all relevant literature was identified (Figure 1).

SEARCH STRATEGY

Studies were only examined if they were published in English. Only peer-reviewed studies were included. No publication date limit was imposed on the search criteria. Search terms included a combination of amputation terms AND gait/biomechanics terms AND secondary disorders. While inclusion for this systematic review did not require the presence of secondary disorders, this term helped to refine the search and identify papers with outcome measures of relevance to the development of secondary disorders in amputee populations. An example search strategy is presented below and a table of the full search strategy, formatted for each database, can be found in Appendix 1.

1) Amputee: "transtibial amput*" OR "transfemoral amput*" OR amput* OR "Lower limb amput*" OR "Lower extremity amput*" OR "Leg prosthesis"
2) Activity: walking OR running OR gait OR locomotion OR biomechanics OR kinematics OR kinetics OR "biomechanical parameter*" OR *symmetr* OR forc* OR angle* OR moment* OR power EMG OR electromyogra*)
3) Secondary disorder: Osteoporosis OR Osteopenia OR "Back Pain" OR Backache OR Osteoarthritis OR "musculoskeletal diseas*" OR "musculoskeletal condition*" OR "secondary diseas*

DATA MANAGEMENT AND SELECTION PROCESS

Records retained for abstract and full paper screening were compiled using an excel spreadsheet designed for systematic reviews [39]. Two reviewers individually applied the eligibility criteria to all records based on

For peer review only - http://bmjopen.bmj.com/site/about/guidelines.xhtml

the inclusion/exclusion criteria outlined in Figure 1. Where conflicts arose, reviewers met to discuss and if agreement still could not be made, a third reviewer was consulted to make the final decision. Review stages progressed from title and abstract review to full paper review (Figure 1). For the title and abstract stage, there were four reviewers, with one person reviewing all papers and the remaining three people each reviewing a third of the papers. For the full paper stage, there were three reviewers, with one person reviewing all papers and the remaining two people reviewing half of the papers each. Level of agreement was assessed using Cohens Kappa [40]. Agreement for the title and abstract review stage was 0.76, while agreement for the full paper review stage was 0.64, where agreement between 0.61-0.80 represents substantial agreement between reviewers. A minimum of five studies that evaluated a specific sub-group were required to be included for evaluation of said sub-group within this systematic review. Due to a limited number of papers included after full text review, studies that examined below ankle amputation (2 papers), rotationplasty amputation (1 paper), bi-lateral amputation (1 paper), osseo-intregration (1 papers), and adult amputees who had an amputation or congenital missing limb as children (0 papers) were ultimately excluded.

CURRENT STAGE

This systematic review is currently at the stage of performing data extraction.

DATA COLLECTION PROCESS

Data is currently being extracted from studies using a standardised excel spreadsheet. All data are being extracted by a single reviewer to ensure consistency, though a random sample of 20% of the data are also being extracted by a second reviewer to assess the risk of bias in the extraction process. Where necessary, extraction from figures is being be performed using the desktop version of WebPlotDigitizer [41], which is a data extraction tool for plots, images and maps.

For peer review only - http://bmjopen.bmj.com/site/about/guidelines.xhtml

DATA ITEMS

Data items being extracted include manuscript title, authors, journal, year, country where data was collected, study type, amputee population, number of participants, amputation level, age, biological sex, body mass, height, time since amputation, cause of amputation, type of prosthetic, years of prosthetic use, secondary symptoms, tasks performed in the study and outcome variables (temporospatial, joint kinematics and joint kinetics). For a detailed list of all biomechanical outcome variables, see Appendix 2. Mean/median values, along with standard deviation/ranges are being extracted. For intervention studies, only the baseline measure will be extracted, thus all data included within this review will be observational and cross-sectional in nature.

During data extraction, it has become evident that some outcome measures may appear very high or very low for both amputee and non-amputee groups within the same study. For example, Hendershot and Wolf [42] examined trunk angle during walking gait using inverse dynamics, identifying that maximum extension for TTA was 4.89˚, TFA was 0.48˚ and non-amputee controls were 2.75˚. Morgenroth, Orendurff [43] also examined trunk angle during walking, however their analysis was based on angle changes of a rigid cluster placed on the 8th thoracic vertebra (T8), with angles relative to the global coordinate system. Thus, they reported that maximum trunk extension of TFA was 26.9˚ while non-amputee controls were 20.5˚. If absolute values were compared, the large maximum angles obtained for TTA's by Morgenroth, Orendurff [43] would drastically alter the differences observed between TTA and TFA across all studies. Therefore, studies which did not examine paired amputee groups (TTA vs TFA or Vascular vs Traumatic) have the potential to drastically alter the results, due to methodological differences in how data were collected. However, if studies recruited both amputees and non-amputees, relative differences compared to non-amputees within the same study could be calculated. Using the example above for Hendershot and Wolf [42], relative maximum trunk angle in TTA was 2.1˚ larger than non-amputee controls and TFA was 2.3˚

For peer review only - http://bmjopen.bmj.com/site/about/guidelines.xhtml

smaller than non-amputee controls, while Morgenroth, Orendurff [43] observed TFA was 6.4˚ larger than non-amputee controls. Unfortunately, if studies only recruited amputees and did not recruit non-amputee controls, calculation of relative differences between amputees and non-amputees cannot be calculated. The diverse range of methodologies included within this review was unexpected and only determinable due to this systematic review collating the largest number of biomechanical gait studies performed on amputees to date. Therefore, to ensure rigorous and objective comparison of outcomes between amputee sub-groups, we have removed 27 studies from screening that did not recruit non-amputee controls (Figure 1), excepting those studies that compared directly between TTA and TFA, or between dysvascular TTA and traumatic TTA. Challenges we are facing during data extraction highlight the key role non-amputee controls play during examination of amputee gait, and therefore, studies wishing to compare their results to prior research should recruit non-amputee participants to facilitate such comparisons.

FUTURE STAGES

All remaining stages of the protocol encompass the future work yet to be started, with major stages including risk of bias assessment and data synthesis.

OUTCOMES AND PRIORITISATION

The primary outcomes will be the biomechanical variables listed in Appendix 2. Reporting of outcome measures will be grouped based on whether previous evidence suggests they may contribute to KOA or LBP. Kinetic measures not already normalised to body mass will be converted to enable comparison between studies. Mean/median outcome measures, relative to controls within the same study, will be compared between amputee groups (TTA vs TFA and Traumatic vs Dysvascular). To directly compare outcome measures between studies for KOA or LBP, measures will be grouped depending on the type of movement: preferred speed flat walking, controlled speed flat walking, preferred speed incline/decline walking, controlled speed incline/decline walking, preferred speed stair climbing or controlled speed stair climbing. These movements were selected as they are commonly performed in daily living and present

For peer review only - http://bmjopen.bmj.com/site/about/guidelines.xhtml

different challenges for amputees. Thus, to examine differences between amputation level, outcome measures related to KOA or LBP will be descriptively compared during each movement, between TTA and TFA, relative to non-amputees. To examine differences between amputation cause, outcome measures related to KOA or LBP will be descriptively compared during each movement, between transtibial traumatic and transtibial dysvascular amputees, relative to non-amputees.

RISK OF BIAS IN INDIVIDUAL STUDIES

Risk of bias will be assessed using the modified Downs and Black method [44, 45]. In this modified version, question 25 which addresses sample size, will be modified to a yes/no question and studies that performed a sample size calculation/power calculation will be awarded one point, while studies without will be awarded zero [44]. Randomised controlled trials will be assessed separately to reduce the impact of increased weighting placed on these studies by the Downs and Black method. Randomised controlled trials will only have baseline outcome measures extracted, so while risk of bias will be analysed separately for observational and intervention studies, outcome measures and presentation of the data will be performed identically across all studies. Two reviewers will both assess each study using the Downs and Black criteria. Where there are conflicts, reviewers will meet to discuss and if they cannot agree, a third reviewer will be consulted to make a final decision.

DATA SYNTHESIS AND DISSEMINATION

The primary goal of this systematic review is to descriptively compare biomechanical risk factors for developing KOA and LBP between amputee sub-groups, irrespective of whether KOA or LBP was present. Due to such a large combination of outcome measures (Appendix 2), sub-groups and gait types, meta-analyses will not be performed. Instead, quantitative results will be synthesised and descriptively compared using biomechanical mean/median values of amputee sub-groups relative to non-amputees. Due to the scope of this review, results are expected to be published in three separate manuscripts: 1) Biomechanical risk factors of KOA between TTA and TFA, relative to non-amputees, 2) Biomechanical risk

For peer review only

factors of LBP between TTA and TFA, relative to non-amputees, and 3) Biomechanical risk factors of KOA and LBP between transtibial amputees with traumatic or dysvascular causes, relative to non-amputees. KOA and LBP will be grouped in the third results paper, as there are far fewer studies that have solely recruited dysvascular amputees. Systematic review analysis and reporting will be performed using the Preferred Reporting Items for Systematic Reviews and Meta-Analyses (PRISMA) guidelines [46].

META-ANALYSIS AND META-BIAS

Due to the high number of movements (e.g. walking, incline walking, decline walking), sub-groups (e.g. TFA, TTA, dysvascular and traumatic amputation) and outcome variables (temporospatial, kinematic and kinetic measures), which significantly reduces the number of studies that are able to be statistically compared for each outcome measure, a meta-analysis will not be performed. Therefore, examination of meta-bias within this review is not possible.

Conclusion

Although there have been several systematic reviews examining development of KOA and LBP in amputees, the number of studies included within these reviews is limited. Furthermore, there have been no comparisons of biomechanical factors leading to KOA and LBP between amputation level, nor between traumatic and dysvascular amputation causes. These sub-groups may experience the development of secondary disorders differently due to altered gait characteristics produced by varying amputation levels and amputation causes. The following three results papers from this systematic review will hopefully illustrate which biomechanical measure are key risk factors for different amputee sub-groups. Understanding these risk factors will hopefully lead to improved personalised rehabilitation programmes and prosthetic designs by physiotherapists, rehabilitation consultants, clinical biomechanists and prosthetists.

For peer review only - http://bmjopen.bmj.com/site/about/guidelines.xhtml

Author Contributions

LW is the guarantor. All authors contributed to conception and design of the study. ES and CM developed the search strategy. LW, MPM, CM and ES contributed to the development of selection criteria and performed study selection. LW drafted the manuscript. All authors read, provided feedback and approved the final manuscript.

Funding Statement

This work was supported by the Engineering and Physical Sciences Research Council, through the RCUK Centre for the Analysis of Motion, Entertainment Research and Applications (CAMERA), Bath, United Kingdom, grant number [EP/M023281/1, EP/T014865/1]. This work was also supported by the Versus Arthritis Centre for Sport Exercise, Exercise and Osteoarthritis Research [Ref 21595].

Conflicts of Interest

The authors declare no conflicts of interest

References

1. Ahmad, N., et al., *The prevalence of major lower limb amputation in the diabetic and non-diabetic population of England 2003–2013.* Diabetes and Vascular Disease Research, 2016. **13**(5): p. 348-353.
2. Sturma, A., L. Hruby, and M. Diers, *Epidemiology and Mechanisms of Phantom Limb Pain*, in *Bionic Limb Reconstruction*, O.C. Aszmann and D. Farina, Editors. 2021, Springer International Publishing: Cham. p. 103-112.
3. Ziegler-Graham, K., et al., *Estimating the Prevalence of Limb Loss in the United States: 2005 to 2050.* Archives of Physical Medicine and Rehabilitation, 2008. **89**(3): p. 422-429.
4. Norvell, D.C., et al., *The prevalence of knee pain and symptomatic knee osteoarthritis among veteran traumatic amputees and nonamputees.* Archives of Physical Medicine and Rehabilitation, 2005. **86**(3): p. 487-493.
5. Struyf, P.A., et al., *The Prevalence of Osteoarthritis of the Intact Hip and Knee Among Traumatic Leg Amputees.* Archives of Physical Medicine and Rehabilitation, 2009. **90**(3): p. 440-446.

For peer review only - http://bmjopen.bmj.com/site/about/guidelines.xhtml

6. Ehde, D.M., et al., *Back pain as a secondary disability in persons with lower limb amputations.* Archives of Physical Medicine and Rehabilitation, 2001. **82**(6): p. 731-734.
7. Stam, H.J., A.-M.V. Dommisse, and H.B.J. Bussmann, *Prevalence of low back pain after transfemoral amputation related to physical activity and other prosthesis-related parameters.* Disability and Rehabilitation, 2004. **26**(13): p. 794-797.
8. Ephraim, P.L., et al., *Phantom Pain, Residual Limb Pain, and Back Pain in Amputees: Results of a National Survey.* Archives of Physical Medicine and Rehabilitation, 2005. **86**(10): p. 1910-1919.
9. Hammarlund, C.S., et al., *Prevalence of back pain, its effect on functional ability and health-related quality of life in lower limb amputees secondary to trauma or tumour: a comparison across three levels of amputation.* Prosthetics and Orthotics International, 2011. **35**(1): p. 97-105.
10. Devan, H., et al., *Exploring Factors Influencing Low Back Pain in People With Nondysvascular Lower Limb Amputation: A National Survey.* PM&R, 2017. **9**(10): p. 949-959.
11. Butowicz, C.M., C.L. Dearth, and B.D. Hendershot, *Impact of Traumatic Lower Extremity Injuries Beyond Acute Care: Movement-Based Considerations for Resultant Longer Term Secondary Health Conditions.* Advances in wound care, 2017. **6**(8): p. 269-278.
12. Wasser, J.G., et al., *Potential lower extremity amputation-induced mechanisms of chronic low back pain: role for focused resistance exercise.* Disability and Rehabilitation, 2019: p. 1-9.
13. Spoden, M., U. Nimptsch, and T. Mansky, *Amputation rates of the lower limb by amputation level – observational study using German national hospital discharge data from 2005 to 2015.* BMC Health Services Research, 2019. **19**(1): p. 8.
14. Imam, B., et al., *Incidence of lower limb amputation in Canada.* Canadian Journal of Public Health, 2017. **108**(4): p. 374-380.
15. Cox, P.S.L., W. SKP, and S. Weaver, *Life after lower extremity amputation in diabetics.* West indian medical journal, 2011. **60**(5): p. 536-540.
16. Gailey, R., et al., *Review of secondary physical conditions associated with lower-limb amputation and long-term prosthesis use.* Journal of Rehabilitation Research & Development, 2008. **45**(1).
17. Devan, H., et al., *Spinal, pelvic, and hip movement asymmetries in people with lower-limb amputation: Systematic review.* Journal of Rehabilitation Research & Development, 2015. **52**: p. 1+.
18. Sivapuratharasu, B., A.M.J. Bull, and A.H. McGregor, *Understanding Low Back Pain in Traumatic Lower Limb Amputees: A Systematic Review.* Archives of Rehabilitation Research and Clinical Translation, 2019. **1**(1): p. 100007.
19. Rosenberg, D.E., et al., *Body mass index patterns following dysvascular lower extremity amputation.* Disability and Rehabilitation, 2013. **35**(15): p. 1269-1275.
20. Lementowski, P.W. and S.B. Zelicof, *Obesity and osteoarthritis.* American Journal of Orthopedics-Belle Mead-, 2008. **37**(3): p. 148.
21. Pezzin, L.E., et al., *Use and satisfaction with prosthetic limb devices and related services 11No commercial party having a direct financial interest in the results of the research supporting this article has or will confer a benefit on the author(s) or on any organization with which the author(s) is/are associated.* Archives of Physical Medicine and Rehabilitation, 2004. **85**(5): p. 723-729.
22. Farrokhi, S., et al., *Biopsychosocial risk factors associated with chronic low back pain after lower limb amputation.* Medical Hypotheses, 2017. **108**: p. 1-9.
23. Farrokhi, S., et al., *A Narrative Review of the Prevalence and Risk Factors Associated With Development of Knee Osteoarthritis After Traumatic Unilateral Lower Limb Amputation.* Military Medicine, 2016. **181**(suppl_4): p. 38-44.
24. Kulkarni, J., et al., *Chronic low back pain in traumatic lower limb amputees.* Clinical Rehabilitation, 2005. **19**(1): p. 81-86.

For peer review only - http://bmjopen.bmj.com/site/about/guidelines.xhtml

25. Oosterhoff, M., J.H.B. Geertzen, and P.U. Dijkstra, *More than half of persons with lower limb amputation suffer from chronic back pain or residual limb pain: a systematic review with meta-analysis.* Disability and Rehabilitation, 2020: p. 1-21.
26. van Eck, C.F. and R.L. McGough, *Clinical outcome of osseointegrated prostheses for lower extremity amputations: a systematic review of the literature.* Current Orthopaedic Practice, 2015. **26**(4).
27. Tomaszewski, P.K., et al., *Numerical analysis of an osseointegrated prosthesis fixation with reduced bone failure risk and periprosthetic bone loss.* Journal of Biomechanics, 2012. **45**(11): p. 1875-1880.
28. Devan, H., et al., *Asymmetrical movements of the lumbopelvic region: Is this a potential mechanism for low back pain in people with lower limb amputation?* Medical Hypotheses, 2014. **82**(1): p. 77-85.
29. Jackson, B.D., et al., *Reviewing knee osteoarthritis — a biomechanical perspective.* Journal of Science and Medicine in Sport, 2004. **7**(3): p. 347-357.
30. Morgenroth, D.C., A.C. Gellhorn, and P. Suri, *Osteoarthritis in the Disabled Population: A Mechanical Perspective.* PM&R, 2012. **4**(5, Supplement): p. S20-S27.
31. Highsmith, M.J., et al., *Low back pain in persons with lower extremity amputation: a systematic review of the literature.* The Spine Journal, 2019. **19**(3): p. 552-563.
32. Iijima, H., et al., *Biomechanical characteristics of stair ambulation in patients with knee OA: A systematic review with meta-analysis toward a better definition of clinical hallmarks.* Gait & Posture, 2018. **62**: p. 191-201.
33. Sagawa, Y., et al., *Biomechanics and physiological parameters during gait in lower-limb amputees: A systematic review.* Gait & Posture, 2011. **33**(4): p. 511-526.
34. Shamseer, L., et al., *Preferred reporting items for systematic review and meta-analysis protocols (PRISMA-P) 2015: elaboration and explanation.* BMJ : British Medical Journal, 2015. **349**: p. g7647.
35. Prinsen, E.C., M.J. Nederhand, and J.S. Rietman, *Adaptation Strategies of the Lower Extremities of Patients With a Transtibial or Transfemoral Amputation During Level Walking: A Systematic Review.* Archives of Physical Medicine and Rehabilitation, 2011. **92**(8): p. 1311-1325.
36. Egloff, C., T. Hügle, and V. Valderrabano, *Biomechanics and pathomechanisms of osteoarthritis.* Swiss Medical Weekly, 2012. **142**(JULY).
37. Davis, E.M., et al., *Longitudinal evidence links joint level mechanics and muscle activation patterns to 3-year medial joint space narrowing.* Clinical Biomechanics, 2019. **61**: p. 233-239.
38. Laird, R.A., et al., *Comparing lumbo-pelvic kinematics in people with and without back pain: a systematic review and meta-analysis.* BMC musculoskeletal disorders, 2014. **15**: p. 229-229.
39. Vonville, H., *Excel workbooks for systematic reviews.* Avaialbe at: https://www. dropbox. com/sh/2q9wh960zfdgc2e/AABc-MEceYlGQMnHwUT8ZWAga, 2015.
40. Cohen, J., *A Coefficient of Agreement for Nominal Scales.* Educational and Psychological Measurement, 1960. **20**(1): p. 37-46.
41. Rohatgi, A. *WebPlotDigitizer - Version 4.5.* 2021; Available from: https://automeris.io/WebPlotDigitizer.
42. Hendershot, B.D. and E.J. Wolf, *Three-dimensional joint reaction forces and moments at the low back during over-ground walking in persons with unilateral lower-extremity amputation.* Clinical Biomechanics, 2014. **29**(3): p. 235-242.
43. Morgenroth, D.C., et al., *The Relationship Between Lumbar Spine Kinematics during Gait and Low-Back Pain in Transfemoral Amputees.* American Journal of Physical Medicine & Rehabilitation, 2010. **89**(8): p. 635-643.
44. Cindy Ng, L.W., et al., *Does exercise training change physical activity in people with COPD? A systematic review and meta-analysis.* Chronic Respiratory Disease, 2011. **9**(1): p. 17-26.

For peer review only - http://bmjopen.bmj.com/site/about/guidelines.xhtml

45. Downs, S.H. and N. Black, *The feasibility of creating a checklist for the assessment of the methodological quality both of randomised and non-randomised studies of health care interventions.* Journal of Epidemiology and Community Health, 1998. **52**(6): p. 377-384.

46. Page, M.J., et al., *PRISMA 2020 explanation and elaboration: updated guidance and exemplars for reporting systematic reviews.* BMJ, 2021. **372**: p. n160.

Figure Legend

Figure 1: Flow chart of paper selection. Exclusion reasons are: 1) no amputees, 2) upper limb amputation, 3) no adult human participants, 4) language not English, 5) review, 6) no quantitative data, 7) paper not found/duplicate, 8) no clinical outcomes, 9) single case study), 10) no results for separate amputee groups, 11) no biomechanical parameters, 12) powered prosthesis only.

For peer review only

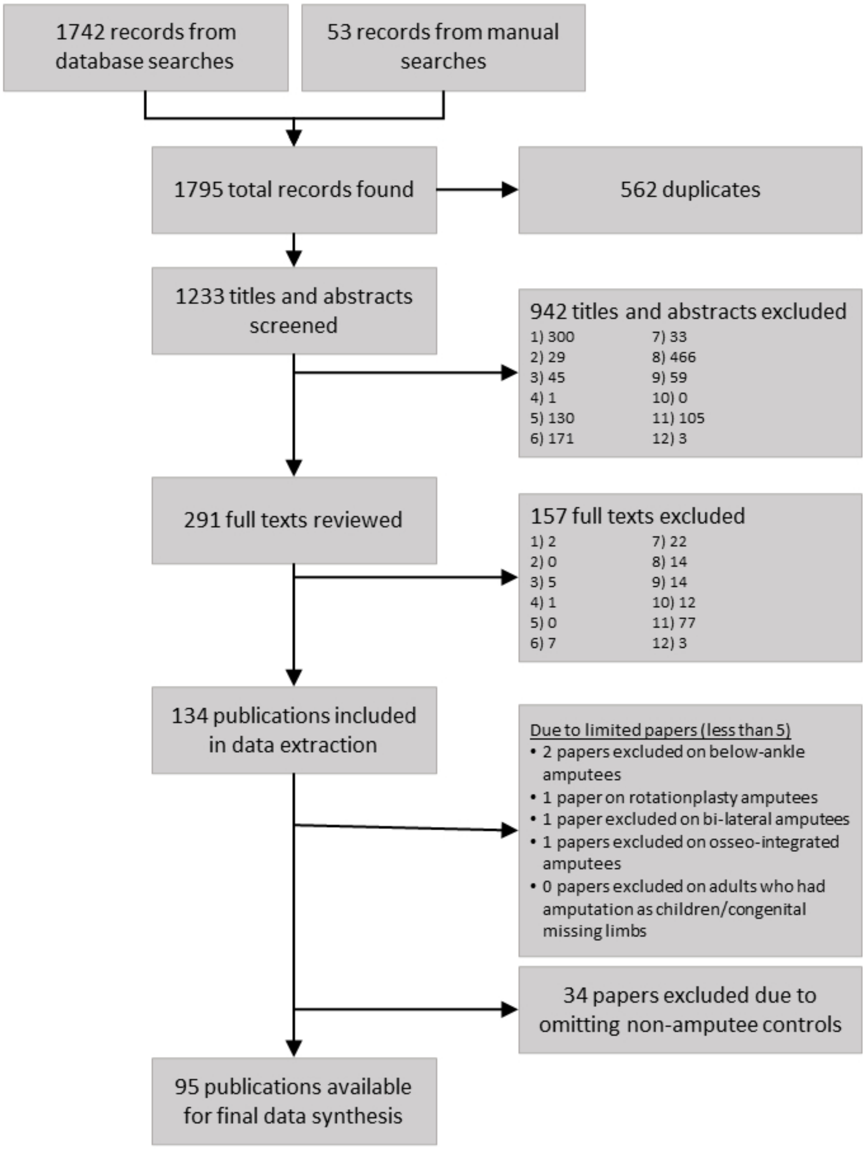

Figure 1: Flow chart of paper selection. Exclusion reasons are: 1) no amputees, 2) upper limb amputation, 3) no adult human participants, 4) language not English, 5) review, 6) no quantitative data, 7) paper not found/duplicate, 8) no clinical outcomes, 9) single case study), 10) no results for separate amputee groups, 11) no biomechanical parameters, 12) powered prosthesis only.

190x250mm (96 x 96 DPI)

Appendix 1: Search strategies for each of the five databases used in this systematic review

Database	keywords
Web of Science	TS=("transtibial amput*" OR "transfemoral amput*" OR amput* OR "Lower limb amput*" OR "Lower extremity amput*" OR "Leg prosthesis") AND TS=(walking OR running OR gait OR locomotion OR biomechanics OR kinematics OR kinetics OR "biomechanical parameter*" OR *symmetr* OR forc* OR angle* OR moment* OR power EMG OR electromyogra*) AND TS=(Osteoporosis OR Osteopenia OR "Back Pain" OR Backache OR Osteoarthritis OR "musculoskeletal diseas*" OR "musculoskeletal condition*" OR "secondary diseas*")<br><br>Limited from 2017-2019
Scopus	(TITLE-ABS-KEY ("transtibial amput*" OR "transfemoral amput*" OR amput* OR "Lower limb amput*" OR "Lower extremity amput*" OR "Leg prosthesis") AND TITLE-ABS-KEY (walking OR running OR gait OR locomotion OR biomechanics OR kinematics OR kinetics OR "biomechanical parameter*" OR *symmetr* OR forc* OR angle* OR moment* OR power OR emg OR electromyogra*) AND TITLE-ABS-KEY (osteoporosis OR osteopenia OR "Back Pain" OR backache OR osteoarthritis OR "musculoskeletal diseas*" OR "musculoskeletal condition*" OR "secondary diseas*")) AND (LIMIT-TO (LANGUAGE , "English") OR LIMIT-TO (LANGUAGE , "German") OR LIMIT-TO (LANGUAGE , "Italian")) AND (LIMIT-TO (PUBYEAR , 2019) OR LIMIT-TO (PUBYEAR , 2018) OR LIMIT-TO (PUBYEAR , 2017))
Pubmed	((("Amputees"[Mesh] OR "Amputation"[Mesh] OR Amput* OR "Amputation, Traumatic"[Mesh] OR "Amputation, Congenital" [Supplementary Concept] OR "lower limb amputation" OR "lower limb amputee" OR "lower extremity amputee" OR “Artificial Limb”[Mesh]) AND ("Walking"[Mesh] OR “Running”[Mesh] OR “Gait”[Mesh] OR gait OR “Locomotion”[Mesh] OR Locomotion OR “Biomechanical Phenomena”[Mesh] OR biomechanic* OR "biomechanical parameter*" OR symmetr* OR angle OR angles OR force OR “Ground Reaction forces” OR power OR “kinetics”[Mesh] OR “Kinematics”[Mesh] OR kinetic* Or kinematic* OR EMG) AND (“Osteoarthritis”[Mesh] OR “osteoporosis”[Mesh] OR osteopenia OR “Back Pain”[Mesh] OR backache OR “musculoskeletal disease*”[Mesh] OR “secondary diseas*” OR “secondary condition” OR “knee osteoarthritis” OR “hip osteoarthritis”))) AND ("2017/07/05"[Date - Publication] : "2019/05/06"[Date - Publication])
Embase	Option A<br>('transtibial amputation'/exp OR 'transtibial amputation' OR 'transfemoral amputation'/exp OR 'transfemoral amputation' OR 'amputee'/exp OR 'amputee' OR 'amputees'/exp OR 'amputees' OR 'individual with amputation'/exp OR 'individual with amputation' OR 'person with amputation'/exp OR 'person with amputation' OR 'artificial leg'/exp OR 'artificial leg' OR 'artificial legs'/exp OR 'artificial legs' OR 'leg prostheses'/exp OR 'leg prostheses' OR 'leg prosthesis'/exp OR 'leg prosthesis' OR 'leg prosthetics'/exp OR 'leg prosthetics' OR 'leg, artificial'/exp OR 'leg, artificial'

For peer review only

	OR 'legs, artificial'/exp OR 'legs, artificial' OR 'lower extremity prosthesis'/exp OR 'lower extremity prosthesis' OR 'lower limb prostheses'/exp OR 'lower limb prostheses' OR 'lower limb prosthesis'/exp OR 'lower limb prosthesis' OR 'prostheses, leg'/exp OR 'prostheses, leg' OR 'prosthesis, leg'/exp OR 'prosthesis, leg' OR 'walking prosthesis'/exp OR 'walking prosthesis' OR 'amputation, traumatic'/exp OR 'amputation, traumatic' OR 'traumatic amputation'/exp OR 'traumatic amputation' OR 'congenital amputation'/exp OR 'congenital amputation') AND ('walking'/exp OR 'walking' OR 'runner'/exp OR 'runner' OR 'running'/exp OR 'running' OR 'kinematics'/exp OR 'kinematics' OR 'human kinetics'/exp OR 'human kinetics' OR 'kinetic analysis'/exp OR 'kinetic analysis' OR 'kinetic mechanism'/exp OR 'kinetic mechanism' OR 'kinetic model'/exp OR 'kinetic model' OR 'kinetics'/exp OR 'kinetics' OR 'biomechanical phenomena'/exp OR 'biomechanical phenomena' OR 'biomechanical phenomenon'/exp OR 'biomechanical phenomenon' OR 'biomechanics'/exp OR 'biomechanics' OR 'biomechanism'/exp OR 'biomechanism' OR 'behavior, locomotor'/exp OR 'behavior, locomotor' OR 'behaviour, locomotor'/exp OR 'behaviour, locomotor' OR 'locomotion'/exp OR 'locomotion' OR 'locomotion pattern'/exp OR 'locomotion pattern' OR 'locomotor activity'/exp OR 'locomotor activity' OR 'locomotor response'/exp OR 'locomotor response' OR 'biped gait'/exp OR 'biped gait' OR 'gait'/exp OR 'gait' OR 'gait analysis'/exp OR 'gait analysis' OR 'gait training'/exp OR 'gait training' OR 'pattern, walking'/exp OR 'pattern, walking' OR 'walking pattern'/exp OR 'walking pattern') AND ('decalcification, pathologic'/exp OR 'decalcification, pathologic' OR 'endocrine osteoporosis'/exp OR 'endocrine osteoporosis' OR 'osteoporosis'/exp OR 'osteoporosis' OR 'osteoporotic decalcification'/exp OR 'osteoporotic decalcification' OR 'osteoarthritis'/exp OR 'osteoarthritis' OR 'back ache'/exp OR 'back ache' OR 'back pain'/exp OR 'back pain' OR 'back pain syndrome'/exp OR 'back pain syndrome' OR 'backache'/exp OR 'backache' OR 'backpain'/exp OR 'backpain' OR 'dorsalgia'/exp OR 'dorsalgia' OR 'pain, back'/exp OR 'pain, back' OR 'musculoskeletal disease'/exp OR 'musculoskeletal disease' OR 'secondary disease'/exp OR 'secondary disease') AND [5-7-2017]/sd NOT [3-5-2019]/sd
CINAHL	OPTION A: Straight search of terms (no subheading selection)<br><br>("transtibial amput*" OR "transfemoral amput*" OR amput* OR "Lower limb amput*" OR "Lower extremity amput*" OR "Leg prosthesis")<br><br>AND<br><br>("Walking" OR “Running” OR “Gait” OR gait OR “Locomotion” OR Locomotion OR “Biomechanical Phenomena” OR biomechanic* OR "biomechanical parameter*" OR symmetr* OR angle OR angles OR force OR “Ground Reaction forces” OR power OR “kinetics” OR “Kinematics” OR kinetic* Or kinematic OR "EMG" OR electromyo*)<br><br>AND

	(“Osteoarthritis” OR “osteoporosis” OR osteopenia OR “Back Pain” OR backache OR “musculoskeletal disease*” OR “secondary diseas*” OR “secondary condition” OR “knee osteoarthritis” OR “hip osteoarthritis”)

For peer review only

Appendix 2: Outcome variable to be extracted from experimental studies

**Temporospatial**	**Kinematics**		**Kinetics**	
BMI	Knee adduction range of motion (ROM)		Peak knee adduction moment (KAM)	
Walking speed	Knee sagittal ROM		KAM loading rate (rate of force development)	
Stride width (from midline)	Hip-knee-ankle adduction angle		KAM impulse	
Stride/step length/symmetry	Varus/valgus angle - knee adduction angle		Peak knee joint contact forces	
Stance/contact time	Knee flexion at heel strike		Joint reaction force at terminal stance	
Leg length discrepancy	Knee flexion at toe off		Peak knee sagittal/flexion/extension plane moments	
Cadence		Knee	Knee rotation moment early stance	
			Knee flexion moment at loading response	
**Ground Reaction Force (GRF)**	Peak hip extension		Flexion moment at terminal stance	
Vertical GRF at heel strike	Peak hip flexion angle		Net work	
Horizontal GRF at heel strike	Hip flexion at toe off		Positive work	
Vertical GRF loading rate	Hip ROM sagittal		Negative work	Knee
Prosthetic horizontal GRF at push-off	Hip flexion at loading response	Hip		
Peak vertical GRF			Peak hip extension moment	Hip
	Peak trunk flexion angle			
	Peak lumbar spine extension		Lumbar-pelvic lateral joint reaction force	
	Lumbar-pelvis spine extension		Anterior lateral joint reaction force - lumbar-pelvic	
	Peak coronal/frontal/lateral/contralateral pelvic tilt		Mediolateral shear joint reaction force of trunk	
	Peak anterior-posterior/sagittal pelvic tilt		Anterior-posterior shear joint reaction force of trunk	
	Pelvic ROM in frontal plane		Compression joint reaction force forces of trunk	
	Pelvic ROM in sagittal plane		Lumbar/pelvic joint power	
	Lumbar transverse plane rotation ROM		Lower back joint contact force	
	Sagittal plane pelvis angle		Joint work L5/S1 (frontal and sagittal plane)	
	Side flexion of trunk-pelvis			Trunk/Pelvis
	Mediolateral trunk sway			
	Lumbar lordosis angle	Trunk/Pelvis		

PRISMA-P (Preferred Reporting Items for Systematic review and Meta-Analysis Protocols) 2015 checklist: recommended items to address in a systematic review protocol*

Section and topic	Item No	Checklist item	Completed
**ADMINISTRATIVE INFORMATION**			
Title:			
Identification	1a	Identify the report as a protocol of a systematic review	Yes
Update	1b	If the protocol is for an update of a previous systematic review, identify as such	N/A
Registration	2	If registered, provide the name of the registry (such as PROSPERO) and registration number	Yes
Authors:			
Contact	3a	Provide name, institutional affiliation, e-mail address of all protocol authors; provide physical mailing address of corresponding author	Yes
Contributions	3b	Describe contributions of protocol authors and identify the guarantor of the review	Yes
Amendments	4	If the protocol represents an amendment of a previously completed or published protocol, identify as such and list changes; otherwise, state plan for documenting important protocol amendments	N/A
Support:			
Sources	5a	Indicate sources of financial or other support for the review	Yes
Sponsor	5b	Provide name for the review funder and/or sponsor	Yes
Role of sponsor or funder	5c	Describe roles of funder(s), sponsor(s), and/or institution(s), if any, in developing the protocol	N/A
**INTRODUCTION**			
Rationale	6	Describe the rationale for the review in the context of what is already known	Yes
Objectives	7	Provide an explicit statement of the question(s) the review will address with reference to participants, interventions, comparators, and outcomes (PICO)	Yes
**METHODS**			
Eligibility criteria	8	Specify the study characteristics (such as PICO, study design, setting, time frame) and report characteristics (such as years considered, language, publication status) to be used as criteria for eligibility for the review	Yes
Information sources	9	Describe all intended information sources (such as electronic databases, contact with study authors, trial registers or other grey literature sources) with planned dates of coverage	Yes
Search strategy	10	Present draft of search strategy to be used for at least one electronic database, including planned limits, such that it could be repeated	Yes

For peer review only

Study records:			
Data management	11a	Describe the mechanism(s) that will be used to manage records and data throughout the review	Yes
Selection process	11b	State the process that will be used for selecting studies (such as two independent reviewers) through each phase of the review (that is, screening, eligibility and inclusion in meta-analysis)	Yes
Data collection process	11c	Describe planned method of extracting data from reports (such as piloting forms, done independently, in duplicate), any processes for obtaining and confirming data from investigators	Yes
Data items	12	List and define all variables for which data will be sought (such as PICO items, funding sources), any pre-planned data assumptions and simplifications	Yes
Outcomes and prioritization	13	List and define all outcomes for which data will be sought, including prioritization of main and additional outcomes, with rationale	Yes
Risk of bias in individual studies	14	Describe anticipated methods for assessing risk of bias of individual studies, including whether this will be done at the outcome or study level, or both; state how this information will be used in data synthesis	Yes
Data synthesis	15a	Describe criteria under which study data will be quantitatively synthesised	Yes
	15b	If data are appropriate for quantitative synthesis, describe planned summary measures, methods of handling data and methods of combining data from studies, including any planned exploration of consistency (such as I^2, Kendall's τ)	N/A
	15c	Describe any proposed additional analyses (such as sensitivity or subgroup analyses, meta-regression)	N/A
	15d	If quantitative synthesis is not appropriate, describe the type of summary planned	N/A
Meta-bias(es)	16	Specify any planned assessment of meta-bias(es) (such as publication bias across studies, selective reporting within studies)	N/A
Confidence in cumulative evidence	17	Describe how the strength of the body of evidence will be assessed (such as GRADE)	Yes

*** It is strongly recommended that this checklist be read in conjunction with the PRISMA-P Explanation and Elaboration (cite when available) for important clarification on the items. Amendments to a review protocol should be tracked and dated. The copyright for PRISMA-P (including checklist) is held by the PRISMA-P Group and is distributed under a Creative Commons Attribution Licence 4.0.**

From: Shamseer L, Moher D, Clarke M, Ghersi D, Liberati A, Petticrew M, Shekelle P, Stewart L, PRISMA-P Group. Preferred reporting items for systematic review and meta-analysis protocols (PRISMA-P) 2015: elaboration and explanation. BMJ. 2015 Jan 2;349(jan02 1):g7647.

For peer review only

BMJ Open

Biomechanical risk factors for knee osteoarthritis and lower back pain in lower limb amputees: protocol for a systematic review

Journal:	*BMJ Open*
Manuscript ID	bmjopen-2022-066959.R1
Article Type:	Protocol
Date Submitted by the Author:	03-Nov-2022
Complete List of Authors:	Wade, Logan; University of Bath, Department for Health<br>McGuigan, M. Polly; University of Bath, Department for Health<br>McKay, Carly; University of Bath, Department for Health<br>Bilzon, James; University of Bath, Department for Health<br>Seminati, Elena; University of Bath, Department for Health
**Primary Subject Heading**:	Research methods
Secondary Subject Heading:	Sports and exercise medicine, Research methods, Reproductive medicine
Keywords:	Diabetic nephropathy & vascular disease < DIABETES & ENDOCRINOLOGY, REHABILITATION MEDICINE, SPORTS MEDICINE

SCHOLARONE™
Manuscripts

BIOMECHANICAL RISK FACTORS FOR KNEE OSTEOARTHRITIS AND LOWER BACK PAIN IN LOWER LIMB AMPUTEES: PROTOCOL FOR A SYSTEMATIC REVIEW

Logan Wade[a,b], M. Polly McGuigan[a,b], Carly McKay[a,c,d], James Bilzon[a,b,d], Elena Seminati[a,b]

a) Department for Health, University of Bath, Bath UK

b) Centre for Analysis of Motion, Entertainment Research and Applications, University of Bath, United Kingdom

c) Centre for Health and Illness and Injury Prevention in Sport, University of Bath, Bath UK

d) Centre for Sport Exercise and Osteoarthritis Research Versus Arthritis, University of Bath, Bath UK

Corresponding Author:

Logan Wade

lw2175@bath.ac.uk

University of Bath

1 West, Office 5.111, Bath, BA2 7AY, United Kingdom

Email:

Logan Wade - lw2175@bath.ac.uk, M. Polly McGuigan - mpm21@bath.ac.uk, Carly McKay - cdm47@bath.ac.uk, James Bilzon - jb438@bath.ac.uk, Elena Seminati - es685@bath.ac.uk

Registration

In accordance with guidelines, this protocol was submitted and approved with the International Prospective Register of Systematic reviews (PROSPERO) on 03/02/2020 and was last updated 21/01/2022 (ID: CRD42020158247).

For peer review only

Abstract

Introduction: There is limited research exploring biomechanical risk factors for the development of knee osteoarthritis (KOA) and lower back pain (LBP) between lower limb amputee sub-groups, [e.g., transtibial amputees (TTA) vs transfemoral amputees (TFA), or TTA dysvascular vs TTA traumatic]. Previous reviews have focussed primarily on studies where symptoms of KOA or LBP are present, however, due to limited study numbers, this hinders their scope and ability to compare between amputee sub-groups. Therefore, the aim of this systematic review is to descriptively compare biomechanical risk factors for developing KOA and LBP between lower limb amputee sub-groups, irrespective of whether KOA or LBP was present.

Methods and analysis: This review is currently in progress and screening results are presented alongside the protocol to highlight challenges encountered during data extraction. Five electronic databases were searched (Medline – Web of Science, PubMed, CINAHL, Embase and Scopus). Eligible studies were observational or interventional, reporting biomechanical gait outcomes for individual legs in adult lower limb amputees during flat walking, incline/decline walking or stair ascent/descent. Two reviewers screened for eligibility and level of agreement was assessed using Cohen's Kappa. Data extraction is ongoing. Risk of bias will be assessed using a modified Downs and Black method, and outcome measures will be descriptively synthesised.

Ethics and dissemination: There are no ethical considerations for this systematic review. Due to its scope, results are expected to be published in three separate manuscripts: 1) Biomechanical risk factors of KOA between TTA and TFA, relative to non-amputees, 2) Biomechanical risk factors of LBP between TTA and TFA, relative to non-amputees, and 3) Biomechanical risk factors of KOA and LBP between transtibial amputees with traumatic or dysvascular causes, relative to non-amputees.

PROSPERO registration number: CRD42020158247).

For peer review only - http://bmjopen.bmj.com/site/about/guidelines.xhtml

Strength and Limitations

- This systematic review protocol follows the Preferred Reporting Items for Systematic Reviews and Meta-Analyses Protocols (PRISMA-P) guidelines.
- Biomechanical gait will be compared between amputee sub-groups (transtibial vs transfemoral amputees, and transtibial dysvascular vs transtibial traumatic amputees).
- Studies must include at least one temporospatial, joint kinematic or joint kinetic outcome measure for individual legs.
- Only amputee studies that included non-amputee controls will be included in the systematic review.

Introduction

Lower limb amputations of the hip, knee and ankle considerably alter walking gait and function, with over 42 000 major lower limb amputations performed over a ten year period (2003-2013) in the UK [1]. In 2005, major lower limb amputations in the USA and UK accounted for over 90% of all major limb amputations [2, 3] and compared to healthy populations, lower-limb amputees have significantly higher rates of secondary disorders such as knee osteoarthritis [4, 5] and lower back pain [6-11]. While there are many biopsychosocial factors that may contribute to the higher rates of secondary disorders, (e.g. mental health, diet, access to facilities or social organisations), the biomechanical factors which result in altered gait of amputee populations will potentially also play a major role [12]. Stable lower limb amputee gait often requires the intact leg to support greater load, which introduces gait asymmetries that over the lifetime, may result in overuse and greater wear of joints and muscles compared to non-amputees. Furthermore, differences between amputation levels (below ankle, below knee and above knee) and amputation causes (traumatic, vascular disease, cancer, congenital) may produce different functional impairments, which could increase the risk of developing knee osteoarthritis (KOA) and lower back pain (LBP) in these different amputee populations.

For peer review only - http://bmjopen.bmj.com/site/about/guidelines.xhtml

For peer review only

Considering the prevalence of lower limb amputations, transfemoral (above the level of the knee) amputees (TFA) and through knee (at the level of the knee joint) amputees account for 17-23% of all amputations [13, 14]. Transtibial (below the level of the knee) amputees (TTA) and through ankle (at the level of the ankle joint) amputees account for 12-32%, while partial foot amputees account for 15-26% of all amputations [13, 14]. Minor amputations of the foot make up the remaining percentages, however these generally do not substantially alter gait and are therefore not a focus of this review. As amputation level moves up the leg, functional mobility and quality of life is reduced [15], requiring greater altered gait mechanics to accommodate the limited power output and instability of the prosthetic limb during stance [12]. Thus, above knee amputees are at an increased risk of developing knee pain [4] and KOA in the intact limb compared to below knee amputees, with OA of the intact knee occurring in roughly 60% of TFAs and 40% of TTAs, compared to just 20% of non-amputees [16]. Similarly, prevalence of LBP is found in roughly 50-76% of lower limb amputees, compared to 35% of non-amputees [10-12]. Evidence suggests that there may not be a difference in prevalence or intensity of LBP between TTA and TFA [17], although a previous systematic review of LBP in lower limb amputees was unable to draw comparisons between TTA and TFA due to limited studies in TTA [18]. Thus, there is a need to explore biomechanical gait differences between TTAs and TFAs, to understand how biomechanical risk factors associated with the development of and potential predisposition to KOA and LBP differ between groups.

While amputation level plays a crucial role in altered gait mechanics, cause of amputation likely also contributes significantly to the development of secondary musculoskeletal symptoms. The two primary causes of amputation are vascular diseases and traumatic accidents, with cancer and congenital causes only making up 1-3% of all amputations [3, 14]. Prevalence of amputation cause varies worldwide, with traumatic amputations making up 6 - 45% of all amputations [3, 14] and patients primarily characterised as being young and fit [3]. Alternatively, dysvascular amputations have increased significantly in recent decades due to the increasing prevalence of diabetes and dysvascular disease, making up 65%-91% of all

For peer review only - http://bmjopen.bmj.com/site/about/guidelines.xhtml

amputations [3, 14]. This population is generally older than other amputee cause types [3] and commonly have a higher body mass index [19], which additionally puts individuals at a greater risk of KOA [20]. Dysvascular amputees also have poorer uptake of prosthetic devices, which further increases their risk of sedentary lifestyle and weight gain after amputation [21]. Counterintuitively, some research suggests that this lower activity status and prosthetic use may result in TFAs having a reduced risk of developing LBP compared to traumatic amputees [16, 18]. Unfortunately, despite a much higher prevalence of dysvascular amputations, gait biomechanics research within this population is relatively limited, especially compared to the high proportion of research surrounding traumatic amputations [4, 11, 18, 22-25]. We therefore need to determine whether current research, investigating the development of secondary disorders primarily in traumatic amputees, is generalisable to dysvascular amputees, and if there are any additional biomechanical factors specific to dysvascular amputees that would increase or decrease their likelihood of developing KOA and LBP.

Additional sub-groups include bi-lateral amputees, osseo-integrated amputees and adult amputees who had an amputation as children or were born without a limb (i.e. congenital amputees). Bilateral amputees have a high variation between individuals, often presenting with multiple amputation levels (e.g. one leg with a trans-tibial amputation and the other with a trans-femoral amputation), which can dramatically alter gait and may influence development of secondary disorders. Osseo-integrated amputees generally do not suffer from skin problems, ill-fitting prosthesis issues or bone degeneration issues of their socket wearing counterparts. Thus, this population may have greater prosthetic use and increased risk of KOA and LBP, although they also have alternate complications such a recurring infections and risk of bone fractures [26, 27]. Finally, adult amputees who experienced amputations during childhood, or were congenital amputees, have spent the most time with their amputation. This group may have altered gait patterns as a function of growing with their prosthesis, which may place them at an increased risk of developing secondary symptoms much earlier in life. Across all amputee sub-groups, the primary barrier to understanding altered biomechanical gait is in recruiting a sufficient sample from each population, especially in these latter specialised sub-groups. Furthermore, longitudinal cohort studies, following patients throughout their life

For peer review only - http://bmjopen.bmj.com/site/about/guidelines.xhtml

are very rare, with most studies being performed cross-sectionally. Therefore, a large-scale systematic review that examines biomechanical gait between amputee sub-groups is presently the best available option for exploring which biomechanical gait factors may contribute to development of KOA or LBP between lower limb amputee populations.

Several reviews have examined amputee biomechanical gait with a focus on KOA and LBP. However, the majority of these reviews have not been performed using systematic methods [11, 22, 23, 28-30], and generally have not described differences between amputee sub-groups, often only including a single sub-group (e.g. only traumatic or transtibial amputees). Moreover, those few systematic reviews on gait and secondary disorders in amputees have generally only been performed on a single amputee subgroup, using studies where symptoms of KOA or LBP are present, which severely limits their scope (11-17 studies per review) and ability to compare between amputee groups [16, 18, 31, 32]. Due to such small study numbers included within these systematic reviews, knowledge of the biomechanical gait characteristics associated with KOA and LBP and their prevalence between amputee sub-groups is considerably limited. Sagawa, Turcot [33] has performed a large-scale systematic review (89 studies) of altered biomechanical gait factors across all lower limb amputees, aiming to broadly characterise biomechanics and physiological parameters during gait. They identified that TTA knee flexion during heel strike is limited to 9-12˚, while TFA knee flexion was zero or negative (extension). Additionally, TFAs had twice the pelvic ROM compared to healthy individuals which may contribute to the development of LBP. Unfortunately, their review was very broad, was not targeted at gait characteristics of KOA and LBP and generally did not make any comparisons or conclusions between sub-groups (e.g., amputation level or amputation cause). To fill this gap in the literature, a large-scale systematic review targeted at identifying how biomechanical risk factors of KOA and LBP differ between amputee sub-groups is needed. Understanding what biomechanical factors influence gait will help facilitate specific and personalised rehabilitation programmes and prosthetic designs.

For peer review only - http://bmjopen.bmj.com/site/about/guidelines.xhtml

OBJECTIVES

While previous systematic reviews have been limited by only including studies with amputees who are diagnosed with KOA and LBP, there is a substantial amount of experimental literature that has examined lower limb amputee gait and posture where no KOA or LBP has been recorded. Because of the high prevalence of KOA and LBP, it is likely that biomechanical abnormalities leading to these secondary disorders will be present across the majority of amputees. Therefore, the aim of this systematic review is to descriptively compare biomechanical risk factors for developing KOA and LBP between amputee sub-groups, irrespective of whether KOA or LBP was present. Amputee sub-groups will be categorised by level of amputation (below ankle, below knee and above knee), cause of amputation (vascular disease, traumatic injury, cancer, congenital) and special sub-groups (bilateral amputees, osseo-integrated amputees, adult amputees who had an amputation or congenital missing limb as children. Individual sub-groups will only be included for analysis if sufficient data is available to support comparisons (see data extraction section).

Methods

This systematic review is currently in progress with the first search complete on 03/07/2017 and a projected end date of 01/12/2023. Screening results are presented within this paper to highlight challenges encountered during data extraction. This approach was chosen to ensure the transparency of our methods and increase the replicability of the review.

ELIGIBILITY CRITERIA

In accordance with PRISMA-P guidelines [34], this protocol was submitted and approved by the International Prospective Register of Systematic reviews (PROSPERO) on 03/02/2020 and was last updated 21/01/2022 (ID: CRD42020158247). This protocol has adhered to the PRIMSA-P guide and checklist for publishing systematic review protocols [34].

For peer review only

STUDY CHARACTERISTICS

Studies included in this review had to be observational studies such as cross-sectional/cohort studies and longitudinal studies. Intervention and randomised control trial (RCT) studies were included in this review but only the control amputee group or baseline measures were extracted (observational data). Review papers, case studies, conference proceedings and animal studies were excluded. Studies that included quantitative biomechanical measures of lower limb amputees were included if results were reported for individual legs (intact leg and prosthetic leg presented separately). To ensure application of valid and thorough biomechanical technique and analysis, data had to include at least one temporospatial, joint kinematic or joint kinetic outcome measure for individual legs (see Appendix 1 for a full list of extracted outcome measures). Outcome variables were determined from previous reviews that outlined biomechanical differences between: amputees and non-amputee populations [12, 17, 22, 23, 28, 33, 35]; healthy non-amputee populations and KOA and LBP non-amputee populations [36-38]; and healthy amputees and amputees with KOA and LBP [12, 16, 18, 31, 32]. While ground reaction force (GRF) outcome measures for individual strides were extracted, studies that only reported GRF measures were not included in this review, as GRF is a measure of full body force and is not specific to the knee joint or lower back region. Observational studies had to be performed during walking on flat, incline or stair surfaces, at either preferred or controlled walking speeds. Studies that only investigated running-specific prostheses or running gaits were not included. Studies that examined powered ankles were included in this review, but only if an unpowered condition was performed. All microprocessor-controlled ankles and knees (devices that do not add energy to the system) were included in this review.

PARTICIPANTS

Lower limb amputees were included in this review, but only if results were separated by different amputation levels (e.g., studies that combined results of transtibial and transfemoral amputees were not included). Due to the differences between child and adult gait, and the focus on development of secondary disorders which primarily occurs in adults, studies performed only on children (younger than 18 years) were not included.

For peer review only - http://bmjopen.bmj.com/site/about/guidelines.xhtml

PATIENT AND PUBLIC INVOLVEMENT

None

INFORMATION SOURCES

Literature searches were performed across five databases: Medline – Web of Science, PubMed, CINAHL, Embase and Scopus. Manual searches were conducted using the reference lists within previous reviews and reference lists within papers obtained from database searches, to ensure all relevant literature was identified (Figure 1).

SEARCH STRATEGY

Studies were only examined if they were published in English. Only peer-reviewed studies were included. No publication date limit was imposed on the search criteria. Search terms included a combination of amputation terms AND gait/biomechanics terms AND secondary disorders. While inclusion for this systematic review did not require the presence of secondary disorders, this term helped to refine the search and identify papers with outcome measures of relevance to the development of secondary disorders in amputee populations. An example search strategy is presented below and a table of the full search strategy, formatted for each database, can be found in Appendix 2.

1) Amputee: "transtibial amput*" OR "transfemoral amput*" OR amput* OR "Lower limb amput*" OR "Lower extremity amput*" OR "Leg prosthesis"
2) Activity: walking OR running OR gait OR locomotion OR biomechanics OR kinematics OR kinetics OR "biomechanical parameter*" OR *symmetr* OR forc* OR angle* OR moment* OR power EMG OR electromyogra*)
3) Secondary disorder: Osteoporosis OR Osteopenia OR "Back Pain" OR Backache OR Osteoarthritis OR "musculoskeletal diseas*" OR "musculoskeletal condition*" OR "secondary diseas*

For peer review only

For peer review only - http://bmjopen.bmj.com/site/about/guidelines.xhtml

DATA MANAGEMENT AND SELECTION PROCESS

Records retained for abstract and full paper screening were compiled using an excel spreadsheet designed for systematic reviews [39]. Two reviewers individually applied the eligibility criteria to all records based on the inclusion/exclusion criteria outlined in Figure 1. Where conflicts arose, reviewers met to discuss and if agreement still could not be made, a third reviewer was consulted to make the final decision. Review stages progressed from title and abstract review to full paper review (Figure 1). For the title and abstract stage, there were four reviewers, with one person reviewing all papers and the remaining three people each reviewing a third of the papers. For the full paper stage, there were three reviewers, with one person reviewing all papers and the remaining two people reviewing half of the papers each. Level of agreement was assessed using Cohens Kappa [40]. Agreement for the title and abstract review stage was 0.76, while agreement for the full paper review stage was 0.64, where agreement between 0.61-0.80 represents substantial agreement between reviewers. A minimum of five studies that evaluated a specific sub-group were required to be included for evaluation of said sub-group within this systematic review. Due to a limited number of papers included after full text review, studies that examined below ankle amputation (2 papers), rotationplasty amputation (1 paper), bi-lateral amputation (1 paper), osseo-intregration (1 papers), and adult amputees who had an amputation or congenital missing limb as children (0 papers) were ultimately excluded.

CURRENT STAGE

This systematic review is currently at the stage of performing data extraction.

DATA COLLECTION PROCESS

Data is currently being extracted from studies using a standardised excel spreadsheet. All data are being extracted by a single reviewer to ensure consistency, though a random sample of 20% of the data are also

For peer review only - http://bmjopen.bmj.com/site/about/guidelines.xhtml

being extracted by a second reviewer to assess the risk of bias in the extraction process. Where necessary, extraction from figures is being be performed using the desktop version of WebPlotDigitizer [41], which is a data extraction tool for plots, images and maps.

DATA ITEMS

Data items being extracted include manuscript title, authors, journal, year, country where data was collected, study type, amputee population, number of participants, amputation level, age, biological sex, body mass, height, time since amputation, cause of amputation, type of prosthetic, years of prosthetic use, secondary symptoms, tasks performed in the study and outcome variables (temporospatial, joint kinematics and joint kinetics). For a detailed list of all biomechanical outcome variables, see Appendix 1. Mean/median values, along with standard deviation/ranges are being extracted. For intervention studies, only the baseline measure will be extracted, thus all data included within this review will be observational and cross-sectional in nature.

During data extraction, it has become evident that some outcome measures may appear very high or very low for both amputee and non-amputee groups within the same study. For example, Hendershot and Wolf [42] examined trunk angle during walking gait using inverse dynamics, identifying that maximum extension for TTA was 4.89˚, TFA was 0.48˚ and non-amputee controls were 2.75˚. Morgenroth, Orendurff [43] also examined trunk angle during walking, however their analysis was based on angle changes of a rigid cluster placed on the 8th thoracic vertebra (T8), with angles relative to the global coordinate system. Thus, they reported that maximum trunk extension of TFA was 26.9˚ while non-amputee controls were 20.5˚. If absolute values were compared, the large maximum angles obtained for TTA's by Morgenroth, Orendurff [43] would drastically alter the differences observed between TTA and TFA across all studies. Therefore, studies which did not examine paired amputee groups (TTA vs TFA or Vascular vs Traumatic) have the potential to drastically alter the results, due to methodological differences in how data were collected.

For peer review only - http://bmjopen.bmj.com/site/about/guidelines.xhtml

However, if studies recruited both amputees and non-amputees, relative differences compared to non-amputees within the same study could be calculated. Using the example above for Hendershot and Wolf [42], relative maximum trunk angle in TTA was 2.1˚ larger than non-amputee controls and TFA was 2.3˚ smaller than non-amputee controls, while Morgenroth, Orendurff [43] observed TFA was 6.4˚ larger than non-amputee controls. Unfortunately, if studies only recruited amputees and did not recruit non-amputee controls, calculation of relative differences between amputees and non-amputees cannot be calculated. The diverse range of methodologies included within this review was unexpected and only determinable due to this systematic review collating the largest number of biomechanical gait studies performed on amputees to date. Therefore, to ensure rigorous and objective comparison of outcomes between amputee sub-groups, we have removed 27 studies from screening that did not recruit non-amputee controls (Figure 1), excepting those studies that compared directly between TTA and TFA, or between dysvascular TTA and traumatic TTA. Challenges we are facing during data extraction highlight the key role non-amputee controls play during examination of amputee gait, and therefore, studies wishing to compare their results to prior research should recruit non-amputee participants to facilitate such comparisons.

FUTURE STAGES

All remaining stages of the protocol encompass the future work yet to be started, with major stages including risk of bias assessment and data synthesis.

OUTCOMES AND PRIORITISATION

The primary outcomes will be the biomechanical variables listed in Appendix 1. Reporting of outcome measures will be grouped based on whether previous evidence suggests they may contribute to KOA or LBP. Kinetic measures not already normalised to body mass will be converted to enable comparison between studies. Mean/median outcome measures, relative to controls within the same study, will be compared between amputee groups (TTA vs TFA and Traumatic vs Dysvascular). To directly compare outcome measures between studies for KOA or LBP, measures will be grouped depending on the type of

For peer review only - http://bmjopen.bmj.com/site/about/guidelines.xhtml

movement: preferred speed flat walking, controlled speed flat walking, preferred speed incline/decline walking, controlled speed incline/decline walking, preferred speed stair climbing or controlled speed stair climbing. These movements were selected as they are commonly performed in daily living and present different challenges for amputees. Thus, to examine differences between amputation level, outcome measures related to KOA or LBP will be descriptively compared during each movement, between TTA and TFA, relative to non-amputees. To examine differences between amputation cause, outcome measures related to KOA or LBP will be descriptively compared during each movement, between transtibial traumatic and transtibial dysvascular amputees, relative to non-amputees.

RISK OF BIAS IN INDIVIDUAL STUDIES

Risk of bias will be assessed using the modified Downs and Black method [44, 45]. In this modified version, question 25 which addresses sample size, will be modified to a yes/no question and studies that performed a sample size calculation/power calculation will be awarded one point, while studies without will be awarded zero [44]. Randomised controlled trials will be assessed separately to reduce the impact of increased weighting placed on these studies by the Downs and Black method. Randomised controlled trials will only have baseline outcome measures extracted, so while risk of bias will be analysed separately for observational and intervention studies, outcome measures and presentation of the data will be performed identically across all studies. Two reviewers will both assess each study using the Downs and Black criteria. Where there are conflicts, reviewers will meet to discuss and if they cannot agree, a third reviewer will be consulted to make a final decision.

DATA SYNTHESIS AND DISSEMINATION

The primary goal of this systematic review is to descriptively compare biomechanical risk factors for developing KOA and LBP between amputee sub-groups, irrespective of whether KOA or LBP was present. Due to such a large combination of outcome measures (Appendix 1), sub-groups and gait types, meta-analyses will not be performed. Instead, quantitative results will be synthesised and descriptively

For peer review only - http://bmjopen.bmj.com/site/about/guidelines.xhtml

compared using biomechanical mean/median values of amputee sub-groups relative to non-amputees. Due to the scope of this review, results are expected to be published in three separate manuscripts: 1) Biomechanical risk factors of KOA between TTA and TFA, relative to non-amputees, 2) Biomechanical risk factors of LBP between TTA and TFA, relative to non-amputees, and 3) Biomechanical risk factors of KOA and LBP between transtibial amputees with traumatic or dysvascular causes, relative to non-amputees. KOA and LBP will be grouped in the third results paper, as there are far fewer studies that have solely recruited dysvascular amputees. The quality of evidence for all outcomes will be judged using the Grading of Recommendations Assessment, Development and Evaluation (GRADE) working group methodology. Systematic review analysis and reporting will be performed using the Preferred Reporting Items for Systematic Reviews and Meta-Analyses (PRISMA) guidelines [46].

META-ANALYSIS AND META-BIAS

Due to the high number of movements (e.g. walking, incline walking, decline walking), sub-groups (e.g. TFA, TTA, dysvascular and traumatic amputation) and outcome variables (temporospatial, kinematic and kinetic measures), which significantly reduces the number of studies that are able to be statistically compared for each outcome measure, a meta-analysis will not be performed. Therefore, examination of meta-bias within this review is not possible.

Author Contributions

LW is the guarantor. LW, MPM, CM, JB and ES contributed to conception and design of the study. ES and CM developed the search strategy. LW, MPM, CM , JB and ES contributed to the development of selection criteria. LW, MPM, CM and ES and performed study selection. LW drafted the manuscript. LW, MPM, CM, JB and ES read, provided feedback and approved the final manuscript.

For peer review only - http://bmjopen.bmj.com/site/about/guidelines.xhtml

Funding Statement

This work was supported by the Engineering and Physical Sciences Research Council, through the RCUK Centre for the Analysis of Motion, Entertainment Research and Applications (CAMERA), Bath, United Kingdom, grant number [EP/M023281/1, EP/T014865/1]. This work was also supported by the Versus Arthritis Centre for Sport Exercise, Exercise and Osteoarthritis Research [Ref 21595].

Conflicts of Interest

The authors declare no conflicts of interest

References

1. Ahmad, N., et al., *The prevalence of major lower limb amputation in the diabetic and non-diabetic population of England 2003–2013.* Diabetes and Vascular Disease Research, 2016. **13**(5): p. 348-353.
2. Sturma, A., L. Hruby, and M. Diers, *Epidemiology and Mechanisms of Phantom Limb Pain*, in *Bionic Limb Reconstruction*, O.C. Aszmann and D. Farina, Editors. 2021, Springer International Publishing: Cham. p. 103-112.
3. Ziegler-Graham, K., et al., *Estimating the Prevalence of Limb Loss in the United States: 2005 to 2050.* Archives of Physical Medicine and Rehabilitation, 2008. **89**(3): p. 422-429.
4. Norvell, D.C., et al., *The prevalence of knee pain and symptomatic knee osteoarthritis among veteran traumatic amputees and nonamputees.* Archives of Physical Medicine and Rehabilitation, 2005. **86**(3): p. 487-493.
5. Struyf, P.A., et al., *The Prevalence of Osteoarthritis of the Intact Hip and Knee Among Traumatic Leg Amputees.* Archives of Physical Medicine and Rehabilitation, 2009. **90**(3): p. 440-446.
6. Ehde, D.M., et al., *Back pain as a secondary disability in persons with lower limb amputations.* Archives of Physical Medicine and Rehabilitation, 2001. **82**(6): p. 731-734.
7. Stam, H.J., A.-M.V. Dommisse, and H.B.J. Bussmann, *Prevalence of low back pain after transfemoral amputation related to physical activity and other prosthesis-related parameters.* Disability and Rehabilitation, 2004. **26**(13): p. 794-797.
8. Ephraim, P.L., et al., *Phantom Pain, Residual Limb Pain, and Back Pain in Amputees: Results of a National Survey.* Archives of Physical Medicine and Rehabilitation, 2005. **86**(10): p. 1910-1919.
9. Hammarlund, C.S., et al., *Prevalence of back pain, its effect on functional ability and health-related quality of life in lower limb amputees secondary to trauma or tumour: a comparison across three levels of amputation.* Prosthetics and Orthotics International, 2011. **35**(1): p. 97-105.
10. Devan, H., et al., *Exploring Factors Influencing Low Back Pain in People With Nondysvascular Lower Limb Amputation: A National Survey.* PM&R, 2017. **9**(10): p. 949-959.

For peer review only - http://bmjopen.bmj.com/site/about/guidelines.xhtml

11. Butowicz, C.M., C.L. Dearth, and B.D. Hendershot, *Impact of Traumatic Lower Extremity Injuries Beyond Acute Care: Movement-Based Considerations for Resultant Longer Term Secondary Health Conditions.* Advances in wound care, 2017. **6**(8): p. 269-278.
12. Wasser, J.G., et al., *Potential lower extremity amputation-induced mechanisms of chronic low back pain: role for focused resistance exercise.* Disability and Rehabilitation, 2019: p. 1-9.
13. Spoden, M., U. Nimptsch, and T. Mansky, *Amputation rates of the lower limb by amputation level – observational study using German national hospital discharge data from 2005 to 2015.* BMC Health Services Research, 2019. **19**(1): p. 8.
14. Imam, B., et al., *Incidence of lower limb amputation in Canada.* Canadian Journal of Public Health, 2017. **108**(4): p. 374-380.
15. Cox, P.S.L., W. SKP, and S. Weaver, *Life after lower extremity amputation in diabetics.* West indian medical journal, 2011. **60**(5): p. 536-540.
16. Gailey, R., et al., *Review of secondary physical conditions associated with lower-limb amputation and long-term prosthesis use.* Journal of Rehabilitation Research & Development, 2008. **45**(1).
17. Devan, H., et al., *Spinal, pelvic, and hip movement asymmetries in people with lower-limb amputation: Systematic review.* Journal of Rehabilitation Research & Development, 2015. **52**: p. 1+.
18. Sivapuratharasu, B., A.M.J. Bull, and A.H. McGregor, *Understanding Low Back Pain in Traumatic Lower Limb Amputees: A Systematic Review.* Archives of Rehabilitation Research and Clinical Translation, 2019. **1**(1): p. 100007.
19. Rosenberg, D.E., et al., *Body mass index patterns following dysvascular lower extremity amputation.* Disability and Rehabilitation, 2013. **35**(15): p. 1269-1275.
20. Lementowski, P.W. and S.B. Zelicof, *Obesity and osteoarthritis.* American Journal of Orthopedics-Belle Mead-, 2008. **37**(3): p. 148.
21. Pezzin, L.E., et al., *Use and satisfaction with prosthetic limb devices and related services 11No commercial party having a direct financial interest in the results of the research supporting this article has or will confer a benefit on the author(s) or on any organization with which the author(s) is/are associated.* Archives of Physical Medicine and Rehabilitation, 2004. **85**(5): p. 723-729.
22. Farrokhi, S., et al., *Biopsychosocial risk factors associated with chronic low back pain after lower limb amputation.* Medical Hypotheses, 2017. **108**: p. 1-9.
23. Farrokhi, S., et al., *A Narrative Review of the Prevalence and Risk Factors Associated With Development of Knee Osteoarthritis After Traumatic Unilateral Lower Limb Amputation.* Military Medicine, 2016. **181**(suppl_4): p. 38-44.
24. Kulkarni, J., et al., *Chronic low back pain in traumatic lower limb amputees.* Clinical Rehabilitation, 2005. **19**(1): p. 81-86.
25. Oosterhoff, M., J.H.B. Geertzen, and P.U. Dijkstra, *More than half of persons with lower limb amputation suffer from chronic back pain or residual limb pain: a systematic review with meta-analysis.* Disability and Rehabilitation, 2020: p. 1-21.
26. van Eck, C.F. and R.L. McGough, *Clinical outcome of osseointegrated prostheses for lower extremity amputations: a systematic review of the literature.* Current Orthopaedic Practice, 2015. **26**(4).
27. Tomaszewski, P.K., et al., *Numerical analysis of an osseointegrated prosthesis fixation with reduced bone failure risk and periprosthetic bone loss.* Journal of Biomechanics, 2012. **45**(11): p. 1875-1880.
28. Devan, H., et al., *Asymmetrical movements of the lumbopelvic region: Is this a potential mechanism for low back pain in people with lower limb amputation?* Medical Hypotheses, 2014. **82**(1): p. 77-85.
29. Jackson, B.D., et al., *Reviewing knee osteoarthritis — a biomechanical perspective.* Journal of Science and Medicine in Sport, 2004. **7**(3): p. 347-357.

For peer review only - http://bmjopen.bmj.com/site/about/guidelines.xhtml

30. Morgenroth, D.C., A.C. Gellhorn, and P. Suri, *Osteoarthritis in the Disabled Population: A Mechanical Perspective.* PM&R, 2012. **4**(5, Supplement): p. S20-S27.
31. Highsmith, M.J., et al., *Low back pain in persons with lower extremity amputation: a systematic review of the literature.* The Spine Journal, 2019. **19**(3): p. 552-563.
32. Iijima, H., et al., *Biomechanical characteristics of stair ambulation in patients with knee OA: A systematic review with meta-analysis toward a better definition of clinical hallmarks.* Gait & Posture, 2018. **62**: p. 191-201.
33. Sagawa, Y., et al., *Biomechanics and physiological parameters during gait in lower-limb amputees: A systematic review.* Gait & Posture, 2011. **33**(4): p. 511-526.
34. Shamseer, L., et al., *Preferred reporting items for systematic review and meta-analysis protocols (PRISMA-P) 2015: elaboration and explanation.* BMJ : British Medical Journal, 2015. **349**: p. g7647.
35. Prinsen, E.C., M.J. Nederhand, and J.S. Rietman, *Adaptation Strategies of the Lower Extremities of Patients With a Transtibial or Transfemoral Amputation During Level Walking: A Systematic Review.* Archives of Physical Medicine and Rehabilitation, 2011. **92**(8): p. 1311-1325.
36. Egloff, C., T. Hügle, and V. Valderrabano, *Biomechanics and pathomechanisms of osteoarthritis.* Swiss Medical Weekly, 2012. **142**(JULY).
37. Davis, E.M., et al., *Longitudinal evidence links joint level mechanics and muscle activation patterns to 3-year medial joint space narrowing.* Clinical Biomechanics, 2019. **61**: p. 233-239.
38. Laird, R.A., et al., *Comparing lumbo-pelvic kinematics in people with and without back pain: a systematic review and meta-analysis.* BMC musculoskeletal disorders, 2014. **15**: p. 229-229.
39. Vonville, H., *Excel workbooks for systematic reviews.* Avaialbe at: https://www. dropbox. com/sh/2q9wh960zfdgc2e/AABc-MEceYlGQMnHwUT8ZWAga, 2015.
40. Cohen, J., *A Coefficient of Agreement for Nominal Scales.* Educational and Psychological Measurement, 1960. **20**(1): p. 37-46.
41. Rohatgi, A. *WebPlotDigitizer - Version 4.5.* 2021; Available from: https://automeris.io/WebPlotDigitizer.
42. Hendershot, B.D. and E.J. Wolf, *Three-dimensional joint reaction forces and moments at the low back during over-ground walking in persons with unilateral lower-extremity amputation.* Clinical Biomechanics, 2014. **29**(3): p. 235-242.
43. Morgenroth, D.C., et al., *The Relationship Between Lumbar Spine Kinematics during Gait and Low-Back Pain in Transfemoral Amputees.* American Journal of Physical Medicine & Rehabilitation, 2010. **89**(8): p. 635-643.
44. Cindy Ng, L.W., et al., *Does exercise training change physical activity in people with COPD? A systematic review and meta-analysis.* Chronic Respiratory Disease, 2011. **9**(1): p. 17-26.
45. Downs, S.H. and N. Black, *The feasibility of creating a checklist for the assessment of the methodological quality both of randomised and non-randomised studies of health care interventions.* Journal of Epidemiology and Community Health, 1998. **52**(6): p. 377-384.
46. Page, M.J., et al., *PRISMA 2020 explanation and elaboration: updated guidance and exemplars for reporting systematic reviews.* BMJ, 2021. **372**: p. n160.

Figure Legend

For peer review only - http://bmjopen.bmj.com/site/about/guidelines.xhtml

Figure 1: Flow chart of paper selection. Exclusion reasons are: 1) no amputees, 2) upper limb amputation, 3) no adult human participants, 4) language not English, 5) review, 6) no quantitative data, 7) paper not found/duplicate, 8) no clinical outcomes, 9) single case study), 10) no results for separate amputee groups, 11) no biomechanical parameters, 12) powered prosthesis only.

For peer review only

For peer review only - http://bmjopen.bmj.com/site/about/guidelines.xhtml

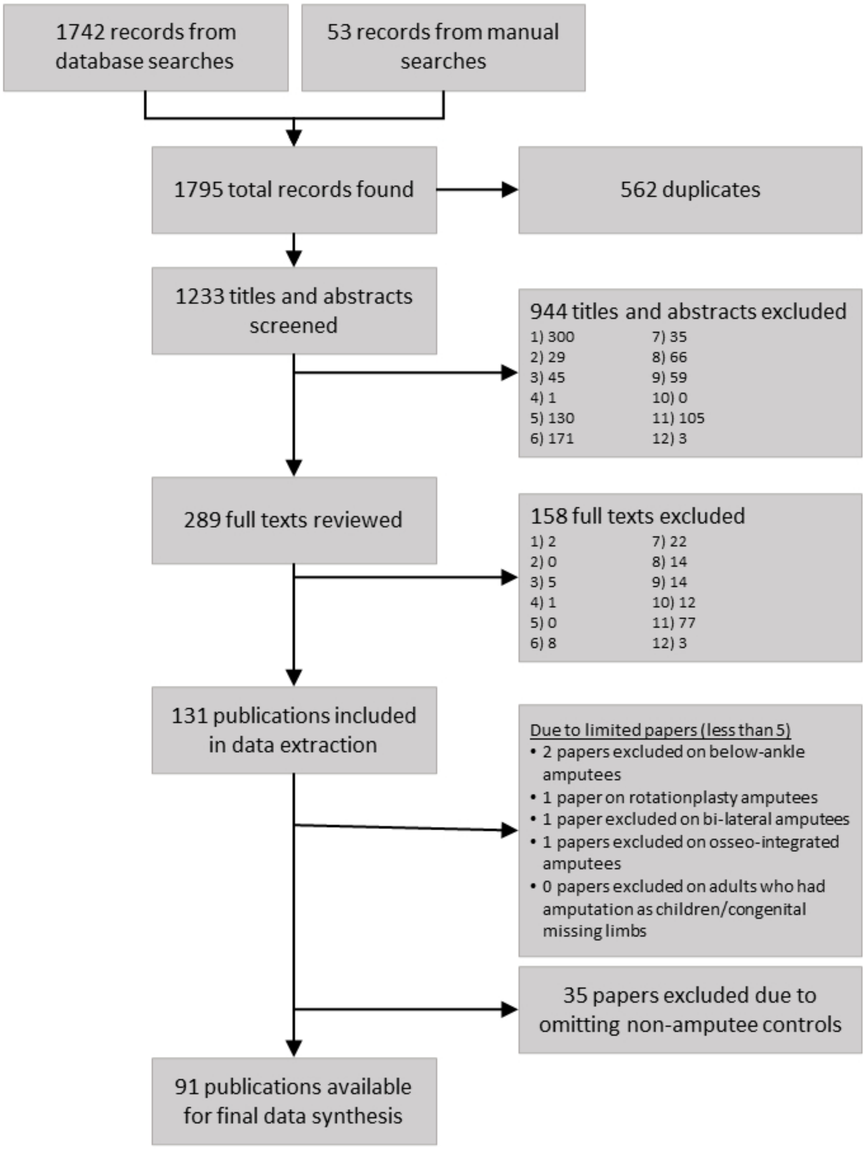

Figure 1: Flow chart of paper selection. Exclusion reasons are: 1) no amputees, 2) upper limb amputation, 3) no adult human participants, 4) language not English, 5) review, 6) no quantitative data, 7) paper not found/duplicate, 8) no clinical outcomes, 9) single case study), 10) no results for separate amputee groups, 11) no biomechanical parameters, 12) powered prosthesis only.

190x250mm (96 x 96 DPI)

Appendix 1: Outcome variable to be extracted from experimental studies

Temporospatial	Kinematics		Kinetics	
BMI	Knee adduction range of motion (ROM)		Peak knee adduction moment (KAM)	
Walking speed	Knee sagittal ROM		KAM loading rate (rate of force development)	
Stride width (from midline)	Hip-knee-ankle adduction angle		KAM impulse	
Stride/step length/symmetry	Varus/valgus angle - knee adduction angle		Peak knee joint contact forces	
Stance/contact time	Knee flexion at heel strike		Joint reaction force at terminal stance	
Leg length discrepancy	Knee flexion at toe off		Peak knee sagittal/flexion/extension plane moments	
Cadence		Knee	Knee rotation moment early stance	
			Knee flexion moment at loading response	
**Ground Reaction Force (GRF)**	Peak hip extension		Flexion moment at terminal stance	
Vertical GRF at heel strike	Peak hip flexion angle		Net work	
Horizontal GRF at heel strike	Hip flexion at toe off		Positive work	
Vertical GRF loading rate	Hip ROM sagittal		Negative work	Knee
Prosthetic horizontal GRF at push-off	Hip flexion at loading response	Hip		
Peak vertical GRF			Peak hip extension moment	Hip
	Peak trunk flexion angle			
	Peak lumbar spine extension		Lumbar-pelvic lateral joint reaction force	
	Lumbar-pelvis spine extension		Anterior lateral joint reaction force - lumbar-pelvic	
	Peak coronal/frontal/lateral/contralateral pelvic tilt		Mediolateral shear joint reaction force of trunk	
	Peak anterior-posterior/sagittal pelvic tilt		Anterior-posterior shear joint reaction force of trunk	
	Pelvic ROM in frontal plane		Compression joint reaction force forces of trunk	
	Pelvic ROM in sagittal plane		Lumbar/pelvic joint power	
	Lumbar transverse plane rotation ROM		Lower back joint contact force	
	Sagittal plane pelvis angle		Joint work L5/S1 (frontal and sagittal plane)	
	Side flexion of trunk-pelvis			Trunk/Pelvis
	Mediolateral trunk sway			
	Lumbar lordosis angle	Trunk/Pelvis		

For peer review only

Appendix 2: Search strategies for each of the five databases used in this systematic review	
Database	keywords
Web of Science	TS=("transtibial amput*" OR "transfemoral amput*" OR amput* OR "Lower limb amput*" OR "Lower extremity amput*" OR "Leg prosthesis") AND TS=(walking OR running OR gait OR locomotion OR biomechanics OR kinematics OR kinetics OR "biomechanical parameter*" OR *symmetr* OR forc* OR angle* OR moment* OR power EMG OR electromyogra*) AND TS=(Osteoporosis OR Osteopenia OR "Back Pain" OR Backache OR Osteoarthritis OR "musculoskeletal diseas*" OR "musculoskeletal condition*" OR "secondary diseas*")
Scopus	(TITLE-ABS-KEY ("transtibial amput*" OR "transfemoral amput*" OR amput* OR "Lower limb amput*" OR "Lower extremity amput*" OR "Leg prosthesis") AND TITLE-ABS-KEY (walking OR running OR gait OR locomotion OR biomechanics OR kinematics OR kinetics OR "biomechanical parameter*" OR *symmetr* OR forc* OR angle* OR moment* OR power OR emg OR electromyogra*) AND TITLE-ABS-KEY (osteoporosis OR osteopenia OR "Back Pain" OR backache OR osteoarthritis OR "musculoskeletal diseas*" OR "musculoskeletal condition*" OR "secondary diseas*")) AND (LIMIT-TO (LANGUAGE , "English") OR LIMIT-TO (LANGUAGE , "German") OR LIMIT-TO (LANGUAGE , "Italian"))
Pubmed	((("Amputees"[Mesh] OR "Amputation"[Mesh] OR Amput* OR "Amputation, Traumatic"[Mesh] OR "Amputation, Congenital" [Supplementary Concept] OR "lower limb amputation" OR "lower limb amputee" OR "lower extremity amputee" OR “Artificial Limb”[Mesh]) AND ("Walking"[Mesh] OR “Running”[Mesh] OR “Gait”[Mesh] OR gait OR “Locomotion”[Mesh] OR Locomotion OR “Biomechanical Phenomena”[Mesh] OR biomechanic* OR "biomechanical parameter*" OR symmetr* OR angle OR angles OR force OR “Ground Reaction forces” OR power OR “kinetics”[Mesh] OR “Kinematics”[Mesh] OR kinetic* Or kinematic* OR EMG) AND (“Osteoarthritis”[Mesh] OR “osteoporosis”[Mesh] OR osteopenia OR “Back Pain”[Mesh] OR backache OR “musculoskeletal disease*”[Mesh] OR “secondary diseas*” OR “secondary condition” OR “knee osteoarthritis” OR “hip osteoarthritis”)))
Embase	Option A<br>('transtibial amputation'/exp OR 'transtibial amputation' OR 'transfemoral amputation'/exp OR 'transfemoral amputation' OR 'amputee'/exp OR 'amputee' OR 'amputees'/exp OR 'amputees' OR 'individual with amputation'/exp OR 'individual with amputation' OR 'person with amputation'/exp OR 'person with amputation' OR 'artificial leg'/exp OR 'artificial leg' OR 'artificial legs'/exp OR 'artificial legs' OR 'leg prostheses'/exp OR 'leg prostheses' OR 'leg prosthesis'/exp OR 'leg prosthesis' OR 'leg prosthetics'/exp OR 'leg prosthetics' OR 'leg, artificial'/exp OR 'leg, artificial' OR 'legs, artificial'/exp OR 'legs, artificial' OR 'lower extremity prosthesis'/exp OR 'lower extremity prosthesis' OR 'lower limb prostheses'/exp OR 'lower limb prostheses' OR 'lower limb prosthesis'/exp OR 'lower limb prosthesis' OR 'prostheses, leg'/exp OR 'prostheses, leg' OR 'prosthesis, leg'/exp OR 'prosthesis, leg' OR 'walking prosthesis'/exp OR 'walking prosthesis' OR 'amputation, traumatic'/exp OR 'amputation, traumatic' OR 'traumatic amputation'/exp OR

	'traumatic amputation' OR 'congenital amputation'/exp OR 'congenital amputation') AND ('walking'/exp OR 'walking' OR 'runner'/exp OR 'runner' OR 'running'/exp OR 'running' OR 'kinematics'/exp OR 'kinematics' OR 'human kinetics'/exp OR 'human kinetics' OR 'kinetic analysis'/exp OR 'kinetic analysis' OR 'kinetic mechanism'/exp OR 'kinetic mechanism' OR 'kinetic model'/exp OR 'kinetic model' OR 'kinetics'/exp OR 'kinetics' OR 'biomechanical phenomena'/exp OR 'biomechanical phenomena' OR 'biomechanical phenomenon'/exp OR 'biomechanical phenomenon' OR 'biomechanics'/exp OR 'biomechanics' OR 'biomechanism'/exp OR 'biomechanism' OR 'behavior, locomotor'/exp OR 'behavior, locomotor' OR 'behaviour, locomotor'/exp OR 'behaviour, locomotor' OR 'locomotion'/exp OR 'locomotion' OR 'locomotion pattern'/exp OR 'locomotion pattern' OR 'locomotor activity'/exp OR 'locomotor activity' OR 'locomotor response'/exp OR 'locomotor response' OR 'biped gait'/exp OR 'biped gait' OR 'gait'/exp OR 'gait' OR 'gait analysis'/exp OR 'gait analysis' OR 'gait training'/exp OR 'gait training' OR 'pattern, walking'/exp OR 'pattern, walking' OR 'walking pattern'/exp OR 'walking pattern') AND ('decalcification, pathologic'/exp OR 'decalcification, pathologic' OR 'endocrine osteoporosis'/exp OR 'endocrine osteoporosis' OR 'osteoporosis'/exp OR 'osteoporosis' OR 'osteoporotic decalcification'/exp OR 'osteoporotic decalcification' OR 'osteoarthritis'/exp OR 'osteoarthritis' OR 'back ache'/exp OR 'back ache' OR 'back pain'/exp OR 'back pain' OR 'back pain syndrome'/exp OR 'back pain syndrome' OR 'backache'/exp OR 'backache' OR 'backpain'/exp OR 'backpain' OR 'dorsalgia'/exp OR 'dorsalgia' OR 'pain, back'/exp OR 'pain, back' OR 'musculoskeletal disease'/exp OR 'musculoskeletal disease' OR 'secondary disease'/exp OR 'secondary disease')
CINAHL	OPTION A: Straight search of terms (no subheading selection)<br><br>("transtibial amput*" OR "transfemoral amput*" OR amput* OR "Lower limb amput*" OR "Lower extremity amput*" OR "Leg prosthesis")<br><br>AND<br><br>("Walking" OR “Running” OR “Gait” OR gait OR “Locomotion” OR Locomotion OR “Biomechanical Phenomena” OR biomechanic* OR "biomechanical parameter*" OR symmetr* OR angle OR angles OR force OR “Ground Reaction forces” OR power OR “kinetics” OR “Kinematics” OR kinetic* Or kinematic OR "EMG" OR electromyo*)<br><br>AND<br><br>(“Osteoarthritis” OR “osteoporosis” OR osteopenia OR “Back Pain” OR backache OR “musculoskeletal disease*” OR “secondary diseas*” OR “secondary condition” OR “knee osteoarthritis” OR “hip osteoarthritis”)

For peer review only

PRISMA-P (Preferred Reporting Items for Systematic review and Meta-Analysis Protocols) 2015 checklist: recommended items to address in a systematic review protocol*

Section and topic	Item No	Checklist item	Completed
**ADMINISTRATIVE INFORMATION**			
Title:			
Identification	1a	Identify the report as a protocol of a systematic review	Yes – Page 1
Update	1b	If the protocol is for an update of a previous systematic review, identify as such	N/A
Registration	2	If registered, provide the name of the registry (such as PROSPERO) and registration number	Yes – Page 1 & 7
Authors:			
Contact	3a	Provide name, institutional affiliation, e-mail address of all protocol authors; provide physical mailing address of corresponding author	Yes – Page 1
Contributions	3b	Describe contributions of protocol authors and identify the guarantor of the review	Yes – Page 14
Amendments	4	If the protocol represents an amendment of a previously completed or published protocol, identify as such and list changes; otherwise, state plan for documenting important protocol amendments	N/A
Support:			
Sources	5a	Indicate sources of financial or other support for the review	Yes – Page 14
Sponsor	5b	Provide name for the review funder and/or sponsor	Yes – Page 14
Role of sponsor or funder	5c	Describe roles of funder(s), sponsor(s), and/or institution(s), if any, in developing the protocol	N/A
**INTRODUCTION**			
Rationale	6	Describe the rationale for the review in the context of what is already known	Yes – Page 3-6
Objectives	7	Provide an explicit statement of the question(s) the review will address with reference to participants, interventions, comparators, and outcomes (PICO)	Yes - Page 6-7
**METHODS**			
Eligibility criteria	8	Specify the study characteristics (such as PICO, study design, setting, time frame) and report characteristics (such as years considered, language, publication status) to be used as criteria for eligibility for the review	Yes – Page 7
Information sources	9	Describe all intended information sources (such as electronic databases, contact with study authors, trial registers or other grey literature sources) with planned dates of coverage	Yes – Page 9
Search strategy	10	Present draft of search strategy to be used for at least one electronic database, including planned limits, such that it could be repeated	Yes – Page 9

For peer review only

Study records:			
Data management	11a	Describe the mechanism(s) that will be used to manage records and data throughout the review	Yes – Page 9-10
Selection process	11b	State the process that will be used for selecting studies (such as two independent reviewers) through each phase of the review (that is, screening, eligibility and inclusion in meta-analysis)	Yes – Page 9-10
Data collection process	11c	Describe planned method of extracting data from reports (such as piloting forms, done independently, in duplicate), any processes for obtaining and confirming data from investigators	Yes – Page 10
Data items	12	List and define all variables for which data will be sought (such as PICO items, funding sources), any pre-planned data assumptions and simplifications	Yes – Page 11
Outcomes and prioritization	13	List and define all outcomes for which data will be sought, including prioritization of main and additional outcomes, with rationale	Yes – Page 12-13
Risk of bias in individual studies	14	Describe anticipated methods for assessing risk of bias of individual studies, including whether this will be done at the outcome or study level, or both; state how this information will be used in data synthesis	Yes – Page 13
Data synthesis	15a	Describe criteria under which study data will be quantitatively synthesised	Yes – Page 13-14
	15b	If data are appropriate for quantitative synthesis, describe planned summary measures, methods of handling data and methods of combining data from studies, including any planned exploration of consistency (such as I^2, Kendall's τ)	N/A
	15c	Describe any proposed additional analyses (such as sensitivity or subgroup analyses, meta-regression)	N/A
	15d	If quantitative synthesis is not appropriate, describe the type of summary planned	N/A
Meta-bias(es)	16	Specify any planned assessment of meta-bias(es) (such as publication bias across studies, selective reporting within studies)	N/A
Confidence in cumulative evidence	17	Describe how the strength of the body of evidence will be assessed (such as GRADE)	Yes – Page 13-14

*** It is strongly recommended that this checklist be read in conjunction with the PRISMA-P Explanation and Elaboration (cite when available) for important clarification on the items. Amendments to a review protocol should be tracked and dated.** **The copyright for PRISMA-P (including checklist) is held by the PRISMA-P Group and is distributed under a Creative Commons Attribution Licence 4.0.**

From: Shamseer L, Moher D, Clarke M, Ghersi D, Liberati A, Petticrew M, Shekelle P, Stewart L, PRISMA-P Group. Preferred reporting items for systematic review and meta-analysis protocols (PRISMA-P) 2015: elaboration and explanation. BMJ. 2015 Jan 2;349(jan02 1):g7647.

For peer review only
